# Anticancer Activities of *Thymus vulgaris* L. in Experimental Breast Carcinoma In Vivo and In Vitro

**DOI:** 10.3390/ijms20071749

**Published:** 2019-04-09

**Authors:** Peter Kubatka, Sona Uramova, Martin Kello, Karol Kajo, Marek Samec, Karin Jasek, Desanka Vybohova, Alena Liskova, Jan Mojzis, Marian Adamkov, Pavol Zubor, Karel Smejkal, Emil Svajdlenka, Peter Solar, Samson Mathews Samuel, Anthony Zulli, Monika Kassayova, Zora Lasabova, Taeg Kyu Kwon, Martin Pec, Jan Danko, Dietrich Büsselberg

**Affiliations:** 1 Department of Medical Biology, Jessenius Faculty of Medicine, Comenius University in Bratislava, 036 01 Martin, Slovakia; pec@jfmed.uniba.sk; 2 Division of Oncology, Biomedical Center Martin, Jessenius Faculty of Medicine, Comenius University in Bratislava, 036 01 Martin, Slovakia; jasek@jfmed.uniba.sk (K.J.); Zora.Lasabova@jfmed.uniba.sk (Z.L.); 3 Department of Obstetrics and Gynecology, Jessenius Faculty of Medicine, Comenius University in Bratislava, 036 01 Martin, Slovakia; sonicka.rybka@gmail.com (S.U.); marek.samec@uniba.sk (M.S.); alenka.liskova@gmail.com (A.L.); Pavol.Zubor@jfmed.uniba.sk (P.Z.); danko@jfmed.uniba.sk (J.D.); 4 Department of Pharmacology, Faculty of Medicine, P. J. Šafarik University, 040 11 Košice, Slovakia; Martin.Kello@upjs.sk (M.K.); 5 St. Elisabeth Oncology Institute, Department of Pathology, 812 50 Bratislava, Slovakia; kkajo@ousa.sk; 6 Biomedical Research Center, Slovak Academy of Sciences, 845 05 Bratislava, Slovakia; 7 Department of Anatomy, Jessenius Faculty of Medicine, Comenius University in Bratislava, 036 01 Martin, Slovakia; desanka.vybohova@jfmed.uniba.sk; 8 Department of Histology and Embryology, Jessenius Faculty of Medicine, Comenius University in Bratislava, 036 01 Martin, Slovakia, adamkov@jfmed.uniba.sk; 9 Department of Natural Drugs, Faculty of Pharmacy, University of Veterinary and Pharmaceutical Sciences, 612 42 Brno, Czech Republic; karel.mejkal@post.cz (K.S.); svajdlenkae@vfu.cz (E.S.); 10 Department of Medical Biology, Faculty of Medicine, P. J. Safarik University, 040 11 Kosice, Slovakia; solarpeter@yahoo.com; 11 Weill Cornell Medicine in Qatar, Qatar Foundation-Education City, Doha 24144, Qatar; sms2016@qatar-med.cornell.edu; 12 Institute for Health and Sport, Victoria University, Melbourne, VIC 3011, Australia; Anthony.Zulli@vu.edu.au; 13 Department of Animal Physiology, Institute of Biology and Ecology, Faculty of Science, P. J. Šafarik University, 040 01 Košice, Slovakia; monika.kassayova@upjs.sk; 14 Department of Immunology and School of Medicine, Keimyung University, Dalseo-Gu, Daegu 426 01, Korea; kwontk@dsmc.or.kr

**Keywords:** angiogenesis, apoptosis, cancer stem cells, cell proliferation, epigenetics, mammary carcinogenesis, MCF-7 cells, MDA-MB-231 cells, predictive and preventive medicine, rat, *Thymus vulgaris*

## Abstract

Naturally-occurring mixtures of phytochemicals present in plant foods are proposed to possess tumor-suppressive activities. In this work, we aimed to evaluate the antitumor effects of *Thymus vulgaris* L. in in vivo and in vitro mammary carcinoma models. Dried *T. vulgaris* (as haulm) was continuously administered at two concentrations of 0.1% and 1% in the diet in a chemically-induced rat mammary carcinomas model and a syngeneic 4T1 mouse model. After autopsy, histopathological and molecular analyses of rodent mammary carcinomas were performed. In addition, in vitro evaluations using MCF-7 and MDA-MB-231 cells were carried out. In mice, *T. vulgaris* at both doses reduced the volume of 4T1 tumors by 85% (0.1%) and 84% (1%) compared to the control, respectively. Moreover, treated tumors showed a substantial decrease in necrosis/tumor area ratio and mitotic activity index. In the rat model, *T. vulgaris* (1%) decreased the tumor frequency by 53% compared to the control. Analysis of the mechanisms of anticancer action included well-described and validated diagnostic and prognostic markers that are used in both clinical approach and preclinical research. In this regard, the analyses of treated rat carcinoma cells showed a CD44 and ALDH1A1 expression decrease and Bax expression increase. Malondialdehyde (MDA) levels and VEGFR-2 expression were decreased in rat carcinomas in both the *T. vulgaris* treated groups. Regarding the evaluations of epigenetic changes in rat tumors, we found a decrease in the lysine methylation status of H3K4me3 in both treated groups (H3K9m3, H4K20m3, and H4K16ac were not changed); up-regulations of miR22, miR34a, and miR210 expressions (only at higher doses); and significant reductions in the methylation status of four gene promoters—ATM serin/threonine kinase, also known as the NPAT gene (ATM); Ras-association domain family 1, isoform A (RASSF1); phosphatase and tensin homolog (PTEN); and tissue inhibitor of metalloproteinase-3 (TIMP3) (the paired-like homeodomain transcription factor (PITX2) promoter was not changed). In vitro study revealed the antiproliferative and proapoptotic effects of essential oils of *T. vulgaris* in MCF-7 and MDA-MB-231 cells (analyses of 3-(4,5-dimethylthiazol-2-yl)-5-(3-carboxymethoxyphenyl)-2-(4-sulfophenyl)-2H-tetrazolium) (MTS); 5-bromo-20-deoxyuridine (BrdU); cell cycle; annexin V/PI; caspase-3/7; Bcl-2; PARP; and mitochondrial membrane potential). *T. vulgaris* L. demonstrated significant chemopreventive and therapeutic activities against experimental breast carcinoma.

## 1. Introduction

It has continuously been demonstrated that dietary plant products (both whole products as well as their isolated compounds) affect the development and progression of cancer disease, including breast cancer (BC) [1,2,3,4,5]. Phytochemicals with antioxidant, anti-inflammatory, and immunomodulatory activity can reduce the growth and spread of cancer [6,7]. In addition, phytochemicals modulate the proliferation, apoptosis, angiogenesis, and other parameters of cancer stem cells [6,7]. The possible anticancer potential of plant bioactive substances is based also on specific targeting of aberrant epigenetic changes which are considered to be hallmarks of carcinogenesis. “Nutri-epigenetics” focuses on the influence of dietary compounds on epigenetic mechanisms including hypermethylation of tumor suppressor gene promoters, aberrant chemical modifications of histones, non-coding RNA (ncRNA)-associated multi-gene silencing, and global DNA hypomethylation [8,9,10]. This approach has gained considerable attention because, in contrast to genetic alterations, epigenetic modifications are reversible and able to affect early carcinogenesis. Several clinical epidemiological studies and meta-analyses have demonstrated that long-term and regular (several times a week) consumption of plant-based whole foods is associated with a reduction in BC risk [11,12,13,14].

*Thymus vulgaris* L. is a herb rich in essential oil and contains oxygenated monoterpenes and monoterpene hydrocarbons as its major chemical components. Specifically, thymol, carvacrol, *p*-cymene, borneol, *trans*-caryophyllene, and *cis*-sabinene hydrate are present at the highest concentrations [15,16]. Furthermore, *Thymus* spp. contain phenolics represented by rosmarinic acid and flavonoid derivatives [17]. These phytochemicals categorize *T. vulgaris* amongst plant foods with the highest antioxidant activity [18]. There are several preclinical studies pointing to the anticancer potential of *T. vulgaris*. For example, the aforementioned herb has demonstrated significant free radical scavenging activity and proapototic effects [19] in the human BC T47D cell line. In a colorectal HCT116 cancer cell model, *T. vulgaris* extract was shown to inhibit proliferation in a concentration- and time-dependent manner [20]. A decrease in proliferation rate has been associated with elevated apoptosis as evidenced by increased caspase-3/7 activity. In addition, *T. vulgaris* decreases the migratory and invasive capacities of HCT116 cells. Tumor inhibitory effects of *T. vulgaris* extract have also been observed against human leukemia THP-1 cells [21]. Finally, *T. vulgaris* essential oil has been observed to significantly inhibit growth of human oral cavity squamous cell carcinoma. This effect is accompanied by the regulation of N-glycan biosynthesis and extracellular signal-regulated kinase 5 (ERK5) and interferon signaling [22].

Anticancer effects of *T. vulgaris* have not been evaluated in a rodent mammary carcinoma model so far. The goal of this study was to evaluate chemopreventive and therapeutic effects of dietary administered *T. vulgaris* using chemically-induced and 4T1 syngeneic breast adenocarcinoma mice and rat models. The rationale of this current study was based on our previous models evaluating anticancer effects of the clove buds, oregano, fruit peel polyphenols, *Chlorella pyrenoidosa*, and young barley [23,24,25,26,27]. These medicinal plants and foods showed significant anticancer effects in experimental mammary carcinogenesis in vivo and in vitro, which were accompanied by proapoptotic, antiproliferative, antiangiogenic, antioxidant, epigenetic, or anti–cancer stem cells (CSCs) effects. As shown previously, mixtures of phytochemicals present in these foods affect a plethora signaling pathways involved in carcinogenesis. We aimed to evaluate more complexly the anticancer effects of *T. vulgaris* against experimental mammary carcinogenesis. Different cancer models—chemoprevention and allograft—were used to define cancer risk reduction (tumor frequency) after long-term administration of *T. vulgaris* or treatment potential (tumor volume) of this plant material, respectively. In addition, we focused on the identification of the mechanisms involved in the anticancer action of *T. vulgaris* in mammary carcinogenesis including representative well-validated parameters of apoptosis (caspase-3, Bax, Bcl-2), proliferation (Ki67), angiogenesis (VEGF, VEGFR-2), oxidative damage (MDA), cancer stem cells (CD24, CD44, ALDH1A1, EpCam), and epigenetics (metylathion status of selected gene promoters, histone chemical modifications, and miRNA expressions). Moreover, some histopathological characteristics of tumors (high/low grade carcinoma ratio) were evaluated. Human cancer cell lines were used to more precisely analyze the mechanism of action (proliferation, cell cycle, and apoptosis) and increased the plausibility of results found in vivo. The linkage between the in vitro and in vivo mechanism of action contributes to more valid results. Moreover, the using of human cancer cells in vitro could improve the extrapolation of results to the human population. Due to the possible differences in cell line genetics, two independent human adenocarcinoma cell lines (MCF-7 and MDA-MB-231) were used.

## 2. Results

### 2.1. Rat Mammary Carcinogenesis and Histopathology of Tumors

*T. vulgaris* (1%, THYME 1 group) significantly inhibited the formation of mammary gland carcinomas in rats by 53% compared to the control (Table 1). In the same experimental group, tumor latency, incidence, and average tumor volume were not changed significantly. The chemopreventive efficacy (tumor frequency) observed in this group was significantly correlated (r = 0.773, *p* = 0.042) to a decrease in tumors. *T. vulgaris* (0.1%, THYME 0.1 group) did not show any significant changes when compared with the control. Cumulative tumor volume, as an additional parameter characterizing the dynamics of tumor volume growth in experimental groups, apparently decreased by 43.5% in the THYME 1 group when compared to the control group.

The mixed papillary/cribriform carcinomas, cribriform carcinomas, and mixed cribriform/papillary carcinomas were the most frequently occurring mammary lesions in this experiment. The rates of high grade (HG)/low grade (LG) carcinomas were not changed after chemoprevention.

### 2.2. Mouse 4T1 Model

**Figure 1:**
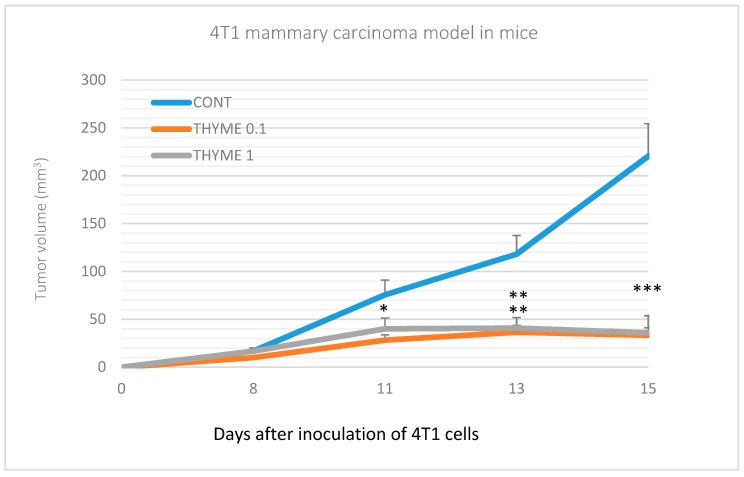
Tumor volume of 4T1 mammary gland cancer in mouse model during the experiment. Data are expressed as mean ± SEM. Significant differences: * *p* < 0.05, ** *p* < 0.01, *** *p* < 0.001 versus CONT.

**Figure 2:**
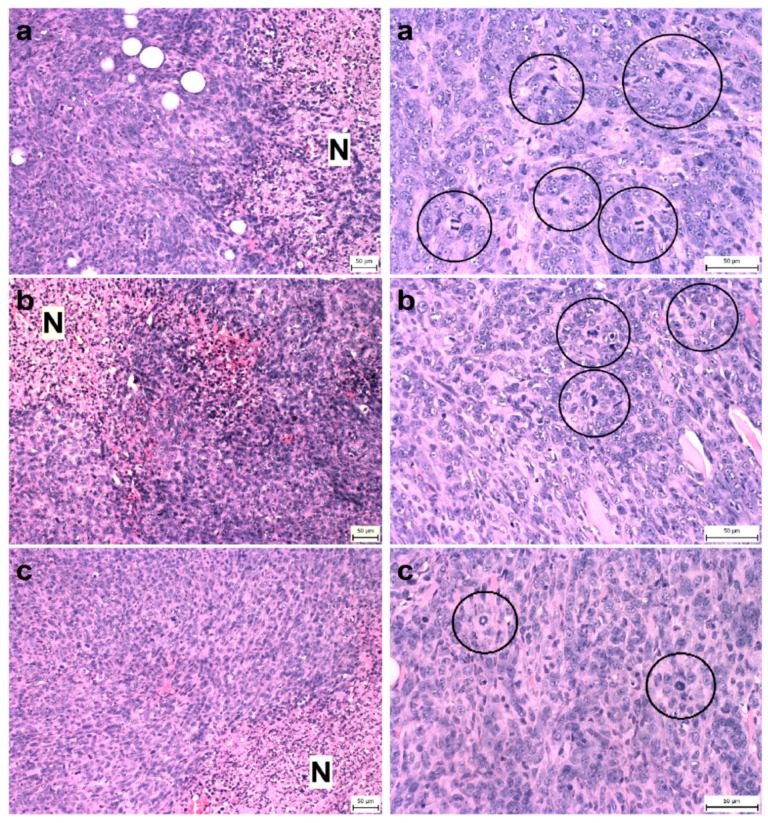
Proportion of necrosis in tumor tissue (N/TT) (left column) and mitotic activity index (MAI) (right column) after treatment with *T. vulgaris* in 4T1 tumors in Balb/c mice. Tumor necrosis (N) mitotic figures (in circles) are showed in experimental groups: (**a**), control group; (**b**), THYME 0.1; (**c**), THYME 1.0; H&E staining magnifications: ×200 (N/TT), ×400 (MAI).

Compared to the control group, *T. vulgaris* significantly (*p* < 0.001) reduced tumor volumes in both treatment groups by 85% (THYME 0.1) and 84% (THYME 1), respectively (Figure 1). Moreover, *T. vulgaris* at both doses significantly (*p* < 0.01) reduced the necrosis/all tumor area ratio by 77% (THYME 0.1) and 81% (THYME 1) versus the control, as well as the mitotic activity index (*p* < 0.05) by 31.5% (THYME 0.1) and 25% (THYME 1) versus the control in 4T1 adenocarcinomas (Figure 2, Table 2).

### 2.3. Immunohistochemistry of Rat Tumors

**Figure 3:**
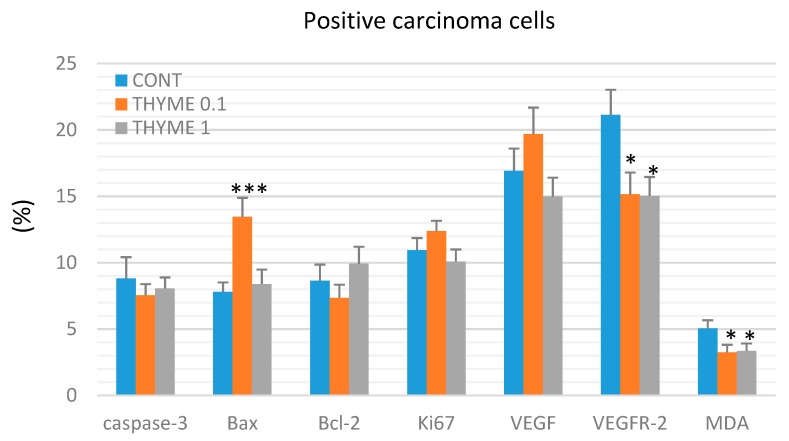
Immunohistochemical evaluation of caspase-3 (cytoplasmic), Bax, Bcl-2, Ki67, VEGFA, VEGFR-2, and MDA expression in rat mammary carcinoma cells after the administration of *T. vulgaris* in two doses. Data are expressed as mean ± SEM. Significant difference, * *p* < 0.05, *** *p* < 0.001 versus CONT. The figure represents the expression of proteins quantified as the average percentage of antigen positive area in standard fields (0.5655 mm^2^) of tumor hotspot areas. The values of protein expression were compared between treated (THYME 0.1, THYME 1) and non-treated (control) carcinoma cells of female rats; at least 60 images for one marker were analyzed.

Figure 3 presents the analyses of apoptosis (cytoplasmic caspase-3, Bax, and Bcl-2), proliferation (Ki67), markers of angiogenesis (VEGF and VEGFR-2), and anti-oxidant effects (based on levels of MDA) in rat mammary carcinoma cells. *T. vulgaris* (THYME 0.1) treatment increased Bax expression by 75.5% (*p* < 0.001) and decreased VEGFR-2 expression by 28% (*p* < 0.05) and MDA levels by 35.5% (*p* < 0.05) versus the control group. *T. vulgaris* (THYME 1) reduced VEGFR-2 expression by 29% (*p* < 0.05) and MDA levels by 33.5% (*p* < 0.05) compared to the control. Caspase-3, Bcl-2, Ki67, and VEGF expressions were not changed significantly in treated carcinoma cells.

**Figure 4:**
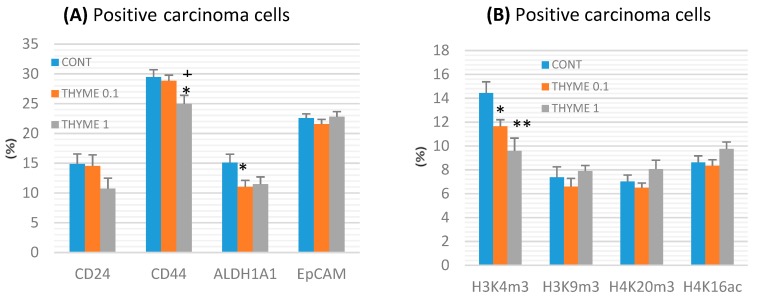
Immunoexpression of cancer stem-cell (**A**) and epigenome (**B**) markers in rat mammary carcinoma cells after treatment with *T. vulgaris*. Data are expressed as mean ± SEM. Significant difference: * *p* < 0.05, ** *p* < 0.01 versus CONT, ^+^ *p* < 0.05 versus THYME 0.1. The values of protein expression were compared between treated (THYME 0.1, THYME 1) and non-treated (control) carcinoma cells of female rats; at least 60 images for one marker were analyzed.

CD44 expression decreased by 15% (*p* < 0.05) in the THYME 1 group and ALDH1A1 expression by 27% (*p* < 0.05) in the THYME 0.1 group versus the control rat carcinoma cells (Figure 4a). A reduction in CD24 expression by 28% (*p* > 0.05) was observed in the THYME 1 group.

Histone 3 and histone 4 chemical modifications showed a significant and dose-dependent decrease of H3K4m3 by 19.5% (*p* < 0.05, THYME 0.1) and 33.5% (*p* < 0.01, THYME 1), respectively, compared to the control (Figure 4b). Changes in H3K9m3, H4K16ac, and H4K20m3 levels in treated groups were not significant.

**Figure 5:**
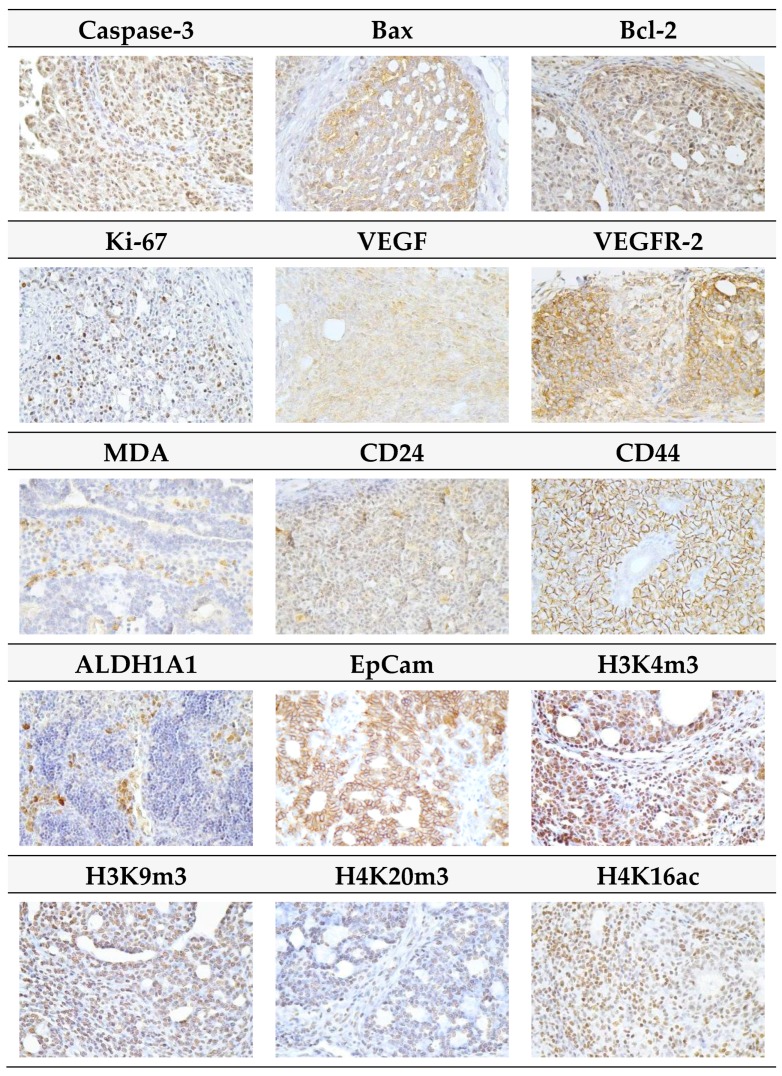
Representative images of expression of caspase-3, Bax, Bcl-2, Ki67, VEGFA, VEGFR-2, MDA, CD24, CD44, ALDH1A1, EpCam, H3K4m3, H3K9m3, H4K20m3, and H4K16ac in rat mammary carcinoma cells. For detection, polyclonal caspase-3 antibody (Bioss, Woburn, MA, USA), polyclonal Bax and Bcl-2 antibodies (Santa Cruz Biotechnology, Paso Robles, CA, USA), monoclonal Ki67 antibody (Dako, Glostrup, Denmark), monoclonal VEGFA and VEGFR-2 antibodies (Santa Cruz Biotechnology, Paso Robles, CA, USA), polyclonal CD24 antibody (GeneTex, Irvine, CA, USA), polyclonal CD44 antibody (Boster, Pleasanton, CA, USA), polyclonal ALDH1A1 antibody (ThermoFisher, Rockford, IL, USA), polyclonal MDA, EpCAM, H3K4m, H3K9m3, and H4K20m3 antibodies (Abcam, Cambridge, MA, USA) and monoclonal H4K16ac antibody (Abcam, Cambridge, MA, USA) were used; final magnifications: ×400.

Representative pictures of the expressions of caspase-3, Bax, Bcl-2, Ki67, VEGFA, VEGFR-2, MDA, CD24, CD44, ALDH1A1, EpCam, H3K4m3, H3K9m3, H4K20m3, and H4K16ac in mammary carcinomas of the rats are outlined in Figure 5.

### 2.4. miRNA Expression

**Figure 6:**
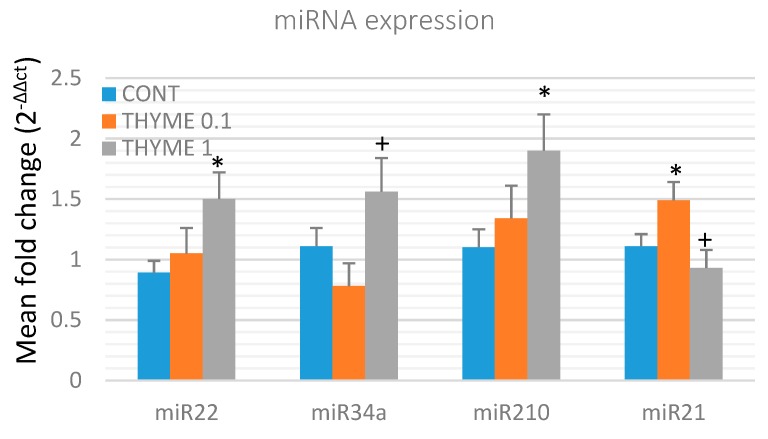
Relative miRNA expression of miR22, miR34a, miR210, and miR21 in rat mammary carcinomas. MiR-191-5p was selected as the internal control miRNA to normalize the cDNA levels of the samples. Data are expressed as mean ± SEM. Significant difference, * *p* < 0.05 versus CONT, ^+^ *p* < 0.05 versus THYME 0.1.

To validate the chemopreventive and therapeutic potential of *T. vulgaris*, we analyzed the expression of several specific miRNAs in rat mammary cancer tissue obtained from in vivo experiments (Figure 6). *T. vulgaris* (THYME 1) significantly increased expression of miR22 by 68.5% (*p* < 0.05) and miR210 by 72.5% (*p* < 0.05) in comparison to the control, and up-regulated miR34a expression by 100% (*p* < 0.05) when compared to the THYME 0.1 group. *T. vulgaris* (THYME 0.1) increased expression of miR21 by 34% (*p* < 0.05) compared to the control group, and *T. vulgaris* (THYME 1) decreased miRNA21 expression by 37.5% (*p* < 0.05) versus The THYME 0.1 group.

### 2.5. Quantitative Methylation Analysis

**Figure 7:**
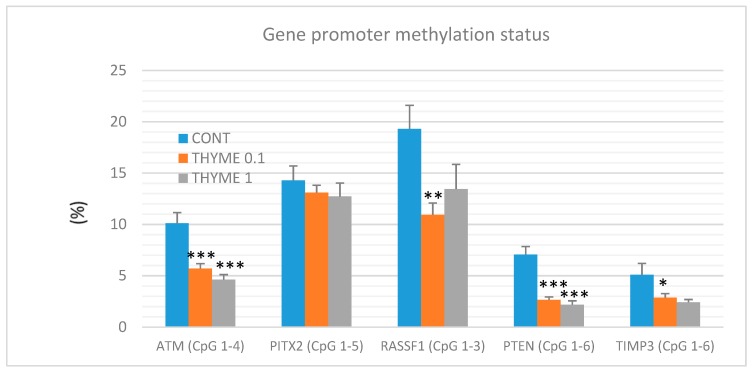
Total DNA promoter methylation status of ATM, PITX2, RASSF1A, PTEN, and TIMP3 genes in rat mammary carcinomas. Total promoter methylation status was taken from all evaluated CpG areas of ATM, PITX2, RASSF1A, PTEN, and TIMP3 in mammary carcinomas in the control and treated groups. Significant difference, * *p* < 0.05, ** *p* < 0.01, *** *p* < 0.001 versus control.

A total of 60 mammary tumor samples of rat mammary carcinomas (from three experimental groups) were evaluated to analyze the methylation status of five gene promoters: ATM including four evaluated CpG (cytosine-phosphate-guanine) sites (CpG 1–4), PITX2 (CpG 1–5), RASSF1 (CpG 1–3), PTEN (CpG 1–6), and TIMP3 (CpG 1–6) (Figure 7). *T. vulgaris* (THYME 0.1) significantly reduced the total methylation status of the ATM promoter by 43% (*p* < 0.001), the RASSF1A promoter by 43.5% (*p* < 0.01), the PTEN promoter by 62.5% (*p* < 0.001), and the TIMP3 promoter by 44% (*p* < 0.05) when compared to the control group. *T. vulgaris* (THYME 1) decreased the methylation status of the ATP promoter by 54.5% (*p* < 0.001), the PTEN promoter by 69.5% (*p* < 0.001) and the TIMP3 promoter by 44% (*p* = 0.06) versus the control. The evaluation promoter methylation status of the TIMP3 gene, the discrepancy in the *p* value and the relatively high decrease of this parameter can be explained by the lower number of data obtained for the THYME 1 group, which was 10 specimens only (the low inducibility of tumors due to a high effectivity of *T. vulgaris* in this group limited our statistical evaluation). A comparison between the control and treated groups did not show any significant differences in the total methylation of the PITX2 promoter. However, a possible (dose-dependent) decrease by 8.5% and 11% was observed (*p* > 0.05).

### 2.6. Physiological in Vivo Effects

*T. vulgaris* (THYME 1) lowered serum levels of low-density lipoprotein (LDL)-cholesterol by 19.1% (*p* < 0.01) versus control animals and by 17% (*p* < 0.05) versus THYME 0.1 animals. Moreover, the *T. vulgaris* (THYME 1) dose significantly lowered total cholesterol levels by 11% (*p* < 0.05) versus THYME 0.1. A significant increase in serum glucose by 8.5% (*p* < 0.05) was observed in the THYME 0.1 group and by 19% (*p* < 0.001) in the THYME 1 group versus the control (data not shown). No differences in final body weight were observed between any groups. A significant increase in food intake was observed: +1.6 g (THYME 0.1) and +2.6 g (THYME 1) versus the control (14.7 g/rat/day). By contrast, in mice, a reduced food intake was found in the THYME 1 group versus the control (1.51 versus 2.10 g/mouse/day). The doses of *T. vulgaris* were safe without undesirable side effects on rats after chronic administration (vitality/activity of animals, hair, mucosa, food and water intake, liver steatosis, hepato/splenomegaly, gastritis, or hematopoietic disorders). In the control group, one animal died immediately after *N*-nitroso-*N*-methylurea (NMU) injection.

### 2.7. In vitro Analyses in MCF-7 and MDA-MB-231 Cells

**Figure 8:**
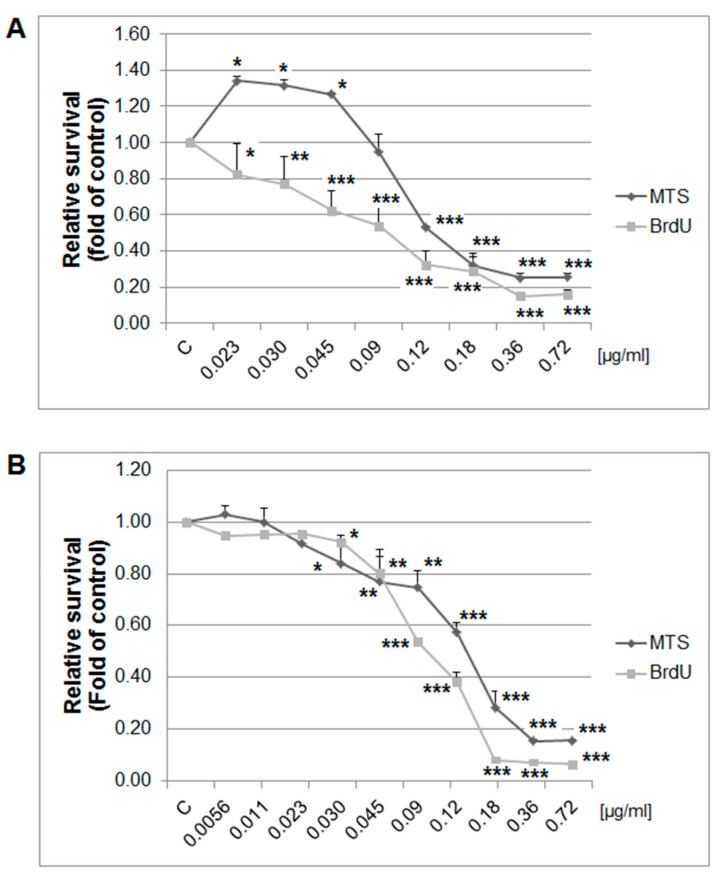
Relative survival of MCF-7 (**A**) and MDA-MB-231 (**B**) cells treated with EOT (0.72–0.023/0.0056 µg/mL) and analyzed by MTS and 5-bromo-20-deoxyuridine (BrdU) incorporation assays. Data were obtained from three independent experiments and significant differences were marked as * *p* < 0.05, ** *p* < 0.01, and *** *p* < 0.001 versus control cells (untreated).

The MTS and BrdU incorporation assays were used to evaluate the possible antiproliferative effects of thyme essential oil (EOT) on MCF-7 and MDA-MB-231 cells. Results showed that EOT significantly decreased in a dose-dependent manner the metabolic activity of cells and subsequently decreased cell survival in both tested cell lines (Figure 8). Depending on analyses, we used different final EOT dilutions (0.13 and 0.12 µg/mL, respectively) for both cell lines for the following experiments. Hormetic effects occurred in the MCF-7 cells at low concentrations of EOT, represented by an increased metabolic activity (not viability). Essential oils could improve the metabolic activity of cancer cells in very low doses and thus provide energy and basic amino acids for synthetic processes. To understand EOT-mediated inhibition of cell growth, BrdU incorporation (with improved informative value compared with MTS) was used to better describe the anti-proliferative activity of EOT.

Flow-cytometric analyses of MCF-7 and MDA-MB-231 cells were performed after 24, 48, and 72 h of treatment with EOT. The evaluation of cell cycle progression (Table 3 and Table 4) after EOT treatment in MCF-7 and MDA-MB-231 cells showed significant differences between these cell lines. In MCF-7 cells, an increased accumulation of cells in the sub-G_0_/G_1_ population (recognized as apoptotic with fractionated DNA) was noted after 24 h to 72 h EOT treatment, with a concomitant decrease of cells in the G1 phase of the cell cycle. On the other hand, cell cycle analyses of MDA-MB-231 cells showed G1 phase cell cycle arrest 24 h after EOT treatment and a decrease of cells in the S phase. Despite the fact that G1 arrest did not last long, EOT treatment increased the accumulation of cells in the sub-G_0_/G_1_ population after 48 h and 72 h.

An increased population of MCF-7 and MDA-MB-231 cells in the sub-G_0_/G_1_ fraction and G1 arrest in MDA-MB-231 cells after EOT treatment suggested an effect on cycle progression and an induction of apoptosis. Annexin V positivity, analyzed as a marker of programmed cell death induction through early membrane changes, showed significant phosphatidyl serine externalization after 24, 48, and 72 h of EOT treatment in both cell lines (Table 5 and Table 6). However, MDA-MB-231 cells showed delayed effects of EOT treatment, followed by a weaker induction of apoptosis. The time span that occurs between the application of EOT and the manifestation of the features typical for the early phase of apoptosis is cell type-dependent. Moreover, the weaker manifestation of apoptosis markers after EOT treatment in MDA-MB-231 cells is in accordance with the G1 cell cycle arrest that delayed exhibition of early membrane changes, followed by phosphatidylserine (PS)-externalization. As annexin V analyses of MDA-MB-231 cells showed, PS-externalization increased in a time-dependent manner, despite G1 cell cycle arrest in 24 h after EOT treatment. Continuous apoptosis and release of MDA-MB-231 cells from the G1 block suggested irreversible changes directed to programmed cell death.

**Figure 9:**
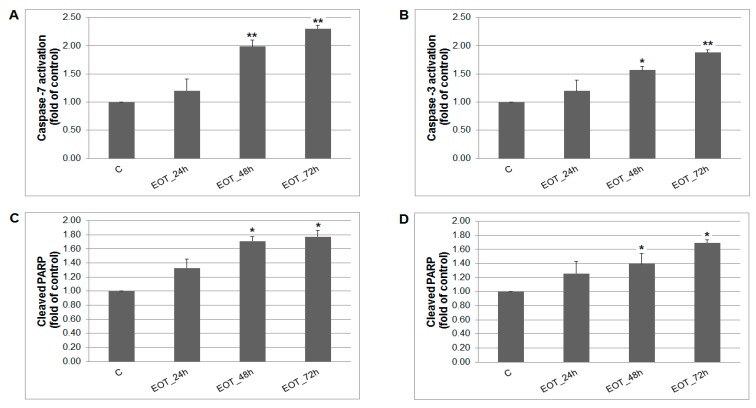
The effect of EOT treatment (0.13 or 0.12 µg/mL) on: (**A**) caspase-7 (MCF-7), (**B**) caspase-3 activation (MDA-MB-231), and PARP cleavage in both cell lines (**C**,**D**) analyzed by flow cytometry. Data were obtained from three independent experiments and significant differences are shown: * *p* < 0.05, ** *p* < 0.01 versus control cells (untreated).

**Figure 10:**
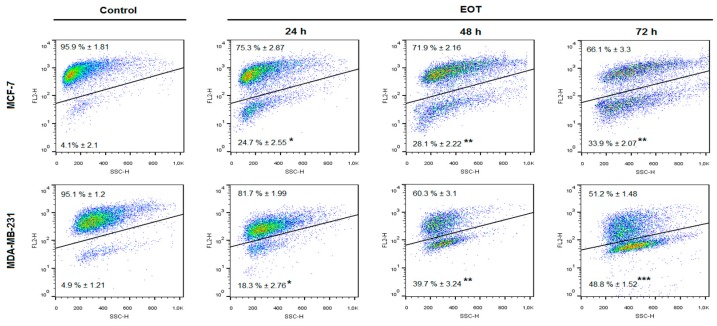
The effects of EOT treatment (0.13 or 0.12 µg/mL) on changes of mitochondrial membrane potential (MMP) in MCF-7 and MDA-MB-231 cells. Data were obtained from three independent experiments and significant differences are shown: * *p* < 0.05, ** *p* < 0.01, *** *p* < 0.001 versus control cells (untreated).

**Figure 11:**
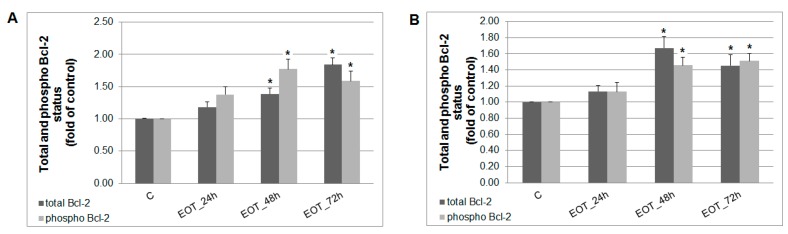
Distribution and activity of anti-apoptotic mitochondria-associated protein Bcl-2 after EOT treatment (0.13 or 0.12 µg/mL) in MCF-7 (**A**) and MDA-MB-231 (**B**) cells analyzed by flow cytometry. Results are expressed as the mean ± SD of three independent experiments and significant differences are given as * *p* < 0.05 versus untreated control.

A caspase-dependent cell death pathway in MCF-7 and MDA-MB-231 cells was confirmed after analysis of caspase-7 or caspase-3 activation (Figure 9). After 48 h of treatment with EOT, MDA-MB-231 cells showed an approximately 0.43 lower increase of caspase activation compared with MCF-7 cells. Moreover, time-dependent depletion of mitochondrial membrane potential (MMP) in both MCF-7 and MDA-MB-231 cells occurred after treatment with EOT (Figure 10). Due to mitochondrial stress after treatment with EOT, a release of anti-apoptotic Bcl-2 (Figure 11) protein complexes from mitochondria to cytosol occurred, which was significant after 48 and 72 h. Analysis of phosphorylation status clearly showed a deactivation in anti-apoptotic activity of Bcl-2 (Figure 11) and confirmed the activation of the mitochondrial apoptosis pathway.

### 2.8. Plant Secondary Metabolites in T. vulgaris

**Figure 12:**
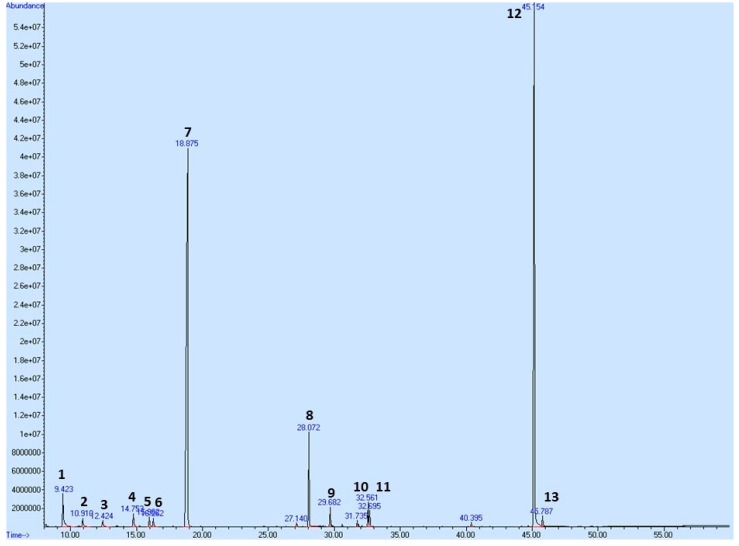
GC/MS chromatogram of EOT with the most abundant peaks. Numbering of peaks is explained in Table 7.

To profile the secondary metabolites of *T. vulgaris* EOT used in this pharmacological study, we performed a Gas Chromatography-Mass Spectrometry (GC-MS) analysis showing its composition (Table 7 and Figure 12). A total of 57 compounds were observed in the oil; however, only compounds exceeding 0.5% of the total content are shown in Table 7, equating to a total of 97.524%. *p*-Cymene and thymol have been identified as major constituents, representing almost 83% of the sum of compounds in *T. vulgaris* essential oil.

## 3. Discussion

The activities of dietary phytochemicals with low toxicity in humans provide a novel approach to cancer treatment, with some undergoing phase I/II clinical trials [28]. To further introduce dietary phytochemicals applicable as novel treatment, we aimed to evaluate the tumor-suppressive activities of *T. vulgaris* in an animal model of breast carcinoma and human carcinoma cell lines.

In this study, the mixture of plant secondary metabolites present in *T. vulgaris* (see Table 7 and Figure 12) demonstrated significant chemopreventive and therapeutic effects against NMU-induced rat mammary carcinogenesis and a mouse 4T1 adenocarcinoma model, respectively. In the NMU model, the greater dose of *T. vulgaris* substantially decreased the most sensitive parameter of cancer chemoprevention model—tumor frequency—by 53% (*p* = 0.044) compared to the control. On the other hand, an increase in tumor frequency by 30% in THYME 0.1 group versus the control group was not significant (*p* = 0.43). Similarly, we found an only non-significant decrease in the average tumor volume and non-significant lengthening in tumor latency in the THYME 0.1 group when compared to the controls. Generally, all non-significant changes in results are considered as a random event in experiments. Hence, we cannot describe the above-mentioned effects of low-dose *T. vulgaris* as pro-cancerogenic, and we can conclude that low-dose *T. vulgaris* was ineffective in cancer risk reduction in the rat chemoprevention study. This statement is not based only on the parameters of rat mammary carcinogenesis, but also on the molecular analyses of apoptosis, proliferation, angiogenesis, oxidative damage, cancer stem cells parameters, histone chemical modifications, methylation of gene promoters, and miRNA expressions of the material obtained from in vivo experiments.

Chemically-induced rat mammary carcinogenesis does not provide the perfect model for the evaluation of the therapeutic effects of drugs and is more specifically suitable for cancer risk evaluation (chemoprevention). Despite the fact that carcinogenesis was induced at the same time in all animals, mammary tumors appeared and grew independently during the whole period of the experiment from 5 to 15 weeks after the application of a carcinogen. In this regard, the experimental group with the highest inducibility of carcinomas (i.e., the control group without therapy) had a greater number of newer tumors with small volumes. On the other hand, groups with effective chemoprevention had relatively a low number of new tumors, and these tumors grew longer and thus may have had relatively larger average volumes. It is therefore difficult to evaluate the crucial parameters of therapeutic effect (tumor volume) in chemocarcinogen-induced mammary carcinogenesis. On the other hand, the allograft model is an appropriate model with which to study tumor volume and the therapeutic effect of a specific drug. Unlike the NMU model, it provides the same (uniform) conditions for all tumors during therapy. Allotransplantation (xenotransplantation) has been used as the treatment experimental approach (model) because the therapeutic hit is directed against the existing cancer cells (we inoculated a confirmed number/10^5^/of viable 4T1 cancer cells per mice) [29,30].

Doses of *T. vulgaris* were chosen according our previous experience with rat models [23,24,25,26,27]. The lower dose of *T. vulgaris* used in this study (0.1%) was approximately two times greater than the common dosage calculated for the Mediterranean population (2 g). In general, rats possess different pharmacokinetics and pharmacodynamics for many drugs compared to humans, and thus greater doses of drugs or dietary phytochemicals are commonly used for assays. Both doses of *T. vulgaris* used were well-tolerated in both the rat and mouse studies. Based on the results discussed below, we deduce that used doses of *T. vulgaris* were correctly chosen and the absorption of active components was efficient. Dietary administered *T. vulgaris* at doses of 0.1 and 1% significantly decreased the tumor volume in mice by 85% and 84% when compared with the untreated controls. Importantly, this result is outstanding as it overcomes the anticancer effects of well-validated synthetic drugs used in BC treatment: manumycin A [29] or paclitaxel [30] in the same 4T1 mouse model. In the chemoprevention study, the higher *T. vulgaris* dose significantly lowered tumor frequency, the most sensitive parameter of rat mammary carcinogenesis, by 53% compared to the control group. Contrary to the mouse model, the reduced dietary dose of *T. vulgaris* was ineffective in the NMU rat model. This result suggests that the sensitivity of different cancer cells in vivo (chemically-induced tumors or 4T1 allografts with different molecular characteristics) to phytochemicals of *T. vulgaris* is dose-dependent.

Our GC-MS analysis showed that the most abundant metabolites in EOT are thymol and *p*-cymene. Based on our in vivo and in vitro results, both molecules could be possible candidates for the clinical management of BC. This is supported by other authors. Most recently, thymol induced toxicity, apoptosis, and cell cycle arrest in MDA-MB231 BC cells [31]. In this study, the evaluations revealed the activation of the intrinsic apoptosis pathway by thymol. Increased reactive oxygen species levels leading to the loss of mitochondrial membrane potential, activation of caspase-3, and DNA damage resulted in S-phase cell cycle arrest. Similarly, the ruthenium(II) *p*-cymene complex significantly inhibited the growth of A17 triple negative BC cells transplanted into Friend leukemia virus B (FVB) syngeneic mice by inhibiting tumor infiltration of regulatory T cells [32]. In another study, ruthenium(II) *p*-cymene complexes induced p53 protein expression in MCF-7cells and reduced their ability to invade [33]. The activation of the p53 pathway after treatment increased mRNA levels of p63, p73, PUMA, BAX, and NOXA genes, while cyclinD1, MMP3, and ID1 gene expressions were significantly decreased. These data, together with the results obtained by our group, demonstrate that thymol, *p*-cymene, and *T. vulgaris* extracts should not be overlooked as possible therapeutics for BC prevention and/or therapy. Importantly, our previous results support the hypothesis that a mixture of phytochemicals with a plethora of biological activities present in whole plant-derived foods could have additive or synergistic effects against BC and could be more effective against cancer than an isolated molecule [7]. Taken together, it is proposed that the simultaneous targeting of multiple (above mentioned) cell signaling pathways induced by thymol, p-cymene, or other phytochemicals comprising the mixtures in plant foods appears to be a more effective biomedical approach. In this regard, numerous pre-clinical and clinical studies have suggested lower effectivity of single phytochemicals compared to whole plant foods against cancer [7]. The chemopreventive effects of various plant foods in a rat mammary carcinoma model have already been described by our group [23,24,25,26,27] and other authors [34,35,36,37].

The mechanism laying behind the chemopreventive effects of *T. vulgaris* was evaluated mainly by the immunohistochemical methods. Programmed cell death can be activated through intrinsic or extrinsic apoptotic pathways. We have assessed mitochondrial mediated apoptosis in vivo. The Bcl-2 family plays a key role in the regulation of this apoptotic pathway; for example, an increased Bax/Bcl-2 ratio up-regulates caspase-3 and increases apoptosis in cancer cells [38]. In our in vivo study, *T. vulgaris* did not induce expression changes in caspase-3, Bcl-2, and Bax after administration of the greater dose of *T. vulgaris*. However, *T. vulgaris* significantly increased Bax expression in rat mammary carcinoma cells only in the low-dose group. However, this result did not correlate with changes in caspase-3 expressions. We propose that low-dose *T. vulgaris* induced the changes and signaling, leading to increased Bax expression in cancer cells in vivo. On the other hand, an organism provides specific defensive mechanisms based on numerous intrinsic/extrinsic signaling pathways which influence the activity and content of the drug in the cell. For example an adaptive increase in degradation enzymes is induced by high doses of the drug. However, the answers to this phenomenon are as yet speculations because there are many compensatory mechanisms that can be activated/suppressed in the cell. We can conclude that apoptosis was not confirmed after *T. vulgaris* treatment in rat tumors in this study (without expression changes in caspase-3 and Bcl-2, and also Bax after a high dose of *T. vulgaris*). On the other hand, our previous studies on chemoprevention, using the same rat model, demonstrated a significant correlation of an increase in Bax/Bcl-2 and caspase-3 expression in mammary carcinoma cells in vivo after dark fruit peel, oregano, and clove bud administration [25,26,27]. Our parallel in vitro study clearly pointed to the proapoptotic potential of *T. vulgaris*, which was demonstrated by the activation of a mitochondria-induced apoptosis pathway in MCF-7 and MDA-MB-231 cells. Specific phytochemicals efficiently raise the Bax/Bcl-2 ratio [39] and initiate the activation of caspase-3 [40] in cancer cells in vitro. Firstly, we found EOT-induced apoptosis in MCF-7 and MDA-MB-231 cell lines by annexin V/PI staining in this study. More specifically, we have shown that increased MMP induced by EOT modulates the activity of key apoptotic regulators. EOT decreased the expression levels of Bcl-2 protein, which caused the release of cytochrome c from mitochondria to the cytoplasm, ultimately resulting in apoptosis [41]. All these changes were associated with an increased expression of execution caspases—caspase-7 (in MCF-7) and caspase-3 (in MDA-MB-231). Consequently, we observed a significant rise in the cleavage of downstream nuclear protein PARP which (through increased DNA fragmentation) induced apoptosis in both cell lines. These processes were linked with an increase in cancer cell population in the sub-G_0_/G_1_ fraction (mainly in MCF-7 cells) and G_1_ phase (in MDA-MB-231 cells) pointing to the block in progression of the cell cycle and the induction of apoptosis. The release of MDA-MB-231 cells from the G_1_ block after 24 h and increased apoptosis in the population suggested irreversible cell damage mediated by EOT treatment. Similar proapoptotic changes (caspase-induced PARP cleavage and the effect on the cell cycle) in different cancer cell lines induced by plant derived extracts were also observed by other authors [42,43]. Our in vitro obtained data clearly pointed to proapototic effects of phytochemicals from *T. vulgaris* in MCF-7 and MDA-MB-231 cells. However, this was not found in the rat BC model in vivo. It is therefore evident that proapototic changes induced by specific phytochemicals are cancer cell line (model)-dependent. *T. vulgaris* compounds differentially target certain signaling pathways according the phenotype (genotype) of the cancer cells. In this study, chemically-induced adenocarcinomas in rats were resistant to proapototic changes and signaling (caspase-3, Bax/Bcl-2) which were observed in MCF-7 and MDA-MB-231 cells after herbal treatment. Our own data [24,25,26,27] as well as data of other authors has clearly demonstrated that plant-derived natural molecules are potential candidates with which to combat cancer through the induction of apoptosis. Phytochemicals (isolated or natural mixtures) can effectively trigger the activation of effector caspases, such as caspase-3 or caspase-7, and increase the Bax/Bcl-2 pro-apoptotic ratio [6]. Nevertheless, the proapoptotic effects of various plant-derived functional foods including *T. vulgaris* will be dependent on the cancer subtype and its molecular characteristics.

Numerous preclinical studies have described the antiproliferative potential of certain phytochemicals in breast carcinogenesis [44,45,46,47,48]. Recently, naturally occurring phytochemicals have gained interest as potential therapeutic BC agents. They directly affect estrogen-dependent and estrogen-independent BC cell proliferation, potentially via affecting important mechanisms, such as COX-2 inhibition or epigenetic modifications, and modulating signaling pathways, such as Hedgehog, NF-κB, Nrf2, PI3 kinase, Plk1, poly-ADP-ribosylation, STAT3, Wnt, and others [49]. Previously, using a chemoprevention BC rat model, we observed a significant decrease in the expression of Ki67 in rat mammary carcinoma cells after young barley, fruit peel polyphenols, oregano, and clove treatment. Our parallel in vitro studies confirmed these results and indicated the significant antiproliferative effects of these plant natural substances in the MCF-7 cell line (using a BrdU proliferation assay, 3-(4,5-dimethylthiazol-2-yl)-2,5-diphenyltetrazolium bromide /MTT/ assay, or cell cycle analysis) [24,25,26,27]. However, *T. vulgaris* in this study did not modulate in vivo expression of Ki67 (an established cell proliferation marker) in rat mammary cancer cells nor grading of tumors (without changes in mitotic activity index or presence of necrosis). However, our in vitro study (using MTS and BrdU assays and cell cycle analysis) clearly confirmed the antiproliferative activity of EOT in estrogen receptor (ER)-positive and ER-negative breast adenocarcinoma cell lines, which is consistent with the results of other authors [50]. Comparing our in vitro and in vivo study, the apoptotic outcomes are similar. We suggest that the antiproliferative activity of phytochemicals present in *T. vulgaris* is cancer cell line/model-dependent. On the other hand, it should be emphasized that we have observed the significant chemoprevention activity of this herb administered in higher doses in rat mammary carcinogenesis. In this regard, the absence of apoptosis and proliferation in rat tumors was counterbalanced by significant positive changes in other parameters of anticancer action observed after *T. vulgaris* treatment (described in detail below). Our results provide a foundation for the trialing of *T. vulgaris* in clinical studies.

Various signaling pathways (e.g., VEGF, EGF, FGF, and HGF) are significantly included in cancer tissue angiogenesis and ensure the heterogeneity of blood vessel structures. The disruption of angiogenic signaling is a growing research area for fighting cancer. VEGF-kinase ligand/receptor signaling plays a crucial role in the formation of new blood vessels [51]. Phytochemicals bind to the VEGFR-tyrosine kinase domain with high affinity, as validated by molecular docking [52]. We have shown that the expression of VEGFR-2, which is an important signal transductor in cells that promotes endothelial tube formation and angiogenesis, was dose-dependently decreased by 28 and 29% in rat tumors in vivo after *T. vulgaris* treatment. This, together with previous results from our laboratory [25,26,27], shows that phytochemicals could have great potential within anti-angiogenic therapy. Targeting the VEGF signaling pathway with plant-derived molecules is a promising option. Unfortunately, despite promising preclinical data for most of the phytochemicals, clinical trials have demonstrated less-convincing results. Future clinical research based on well-organized and randomized studies with placebo controls is strongly warranted.

The imbalance between electrophilic oxidative stress and protective biomolecular nucleophiles can produce aberrant redox homeostasis, resulting in increased oxidative stress and mutagenesis. Consequently, these changes can cause numerous pathologies, including cancer [53]. Antioxidant activities of phytochemicals in the cells are well-described [54]. We have demonstrated that mixtures of phytochemicals present in whole foods such as young barley (rich in flavonoids) or clove buds (rich in phenolics and terpenoids) significantly reduce the oxidative damage of cellular proteins and lipids [26,27]. MDA is often used as a marker of oxidative damage of lipids by free radicals [55]. In this study, *T. vulgaris* decreased the production of MDA in rat cancer cells in vivo. These data indicate the antioxidant potential of *T. vulgaris* in rat carcinomas and point to a proposed geno-protective mechanism of action in carcinogenesis. However, results of in vitro studies affirming anti- and even pro-oxidant activities of different classes of phytochemicals have been commonly related to high doses used, which do not mimic human cellular physiology and possible real intake [56].

Cancer stem cells (CSCs) are a small subset of cancer cell mass, which is responsible for the initiation, promotion, and progression of carcinogenesis. It is well-documented that CSCs may be responsible for resistance to chemotherapy, which leads to relapse and multidrug resistance in cancer disease [57]. Concerning chemoprevention, it is supposed that phytochemicals improve efficacy against carcinoma cells through a CSC-associated mechanism. In clinical practice, clear criteria for the identification of breast CSCs have yet to be established. However, several markers have been introduced. Phenotype CD44^+^/CD24^−/low^ and upregulation of CD24, ALDH1, EpCAM/ESA, and nestin demonstrate worsened prognosis of BC [55]. CD24, CD44, EpCam, and ALDH1A1 have the functions of self-renewal, tumorigenicity, differentiation, proliferation, adhesion, development, clonogenicity, reprogramming, and progression of BC cells [58]. Regarding the mechanism of action, the CD44^+^/CD24^−^ phenotype is linked to an increase of the PI3K/Akt/mTOR-signaling pathway in MCF-7 cells, and ERK activation is a key event in EGFR-dependent regulation of CD44^+^/CD24^−^ population in triple-negative BC cells [59,60]. CD44 activates hypoxia-inducible HIF-1α signaling via the ERK pathway, and this signaling is responsible for increased cancer cell viability and motility under hypoxic conditions [61]. EpCAM modulates NF-κB transcription factor activity and IL-8 expression in MDA-MB-231 and MCF-7 cells. This observation confirms the role of EpCAM signaling in the modulating of BC invasion [62]. Finally, the suppression of the HER2/Akt signaling pathway in HER2-positive BC is linked to the down-regulation of ALDH1 activity and the eradication of the cancer stem-like population [63]. ALDH1A1 activity is also associated with ERK→C/EBPβ [64] and NOTCH [65] signaling. With reference to our study, CD24, CD44, EpCam, and ALDH1A1 have been previously proven to be good CSC markers in chemocarcinogen-induced rat carcinogenesis [66,67]. This study aimed to characterize *T. vulgaris* effects on the expression of CSC-like markers in the chemoprevention model of breast carcinogenesis and a significant decrease in CD44 and ALH1A1 expression in rat mammary tumors was found. Our recent analyses have demonstrated positive downregulation of CD24 and EpCAM expression after oregano administration [26] and decreased expression of CD24 and CD44 [27] after treatment with clove buds in chemically-induced mammary adenocarcinomas in rats. Data from our experiments clearly points to the beneficial effects of oregano, cloves, and *T. vulgaris* on clinically well-validated markers of CSCs in the breast. However, analyses of the specific mechanisms responsible for the anti-CSC effects of whole plant foods require more complex scientific investigation, including elucidation of the involved cell signaling pathways.

Phytochemicals demonstrate apparent anticancer potential by specifically targeting aberrant epigenetic changes [68]. Cancer risk prediction is rapidly continuing to evolve in medicinal practice. In this regard, clinical research can define specific epigenetic modulations, including the global methylation status of oncogenes and tumor-suppressor genes, histone chemical modifications, and non-coding RNA-associated multi-gene controls [69]. All evaluated epigenetic markers that were chosen are well-described and validated diagnostic and prognostic measures that are used in both clinical approaches and rodent models. Aberrant histone modifications and changed expression of histone-modifying enzymes may be caused by genetic mutations in chromatin regulatory enzymes and by epigenetic changes. Dietary phytochemicals are able to modulate epigenetic modifications and thus are actively involved in chemoprevention [70]. The role of plant natural substances in histone methylation and acetylation patterns has been documented in several in vitro and in vivo studies [71,72,73,74]. Alterations in methylation and acetylation of histone lysine residues, such as H3K4m3, H3K9m3, H4K20m3, and H4K16ac, have been frequently associated with breast carcinoma [75,76]. *T. vulgaris* significantly and dose-dependently lowered H3K40m3 levels in this study. Similarly, recent results from our group have shown that dietary administered clove buds significantly and in a dose-dependent manner increase the levels of H4K20m3 and H4K16ac in chemically-induced rat mammary cancer [27]. Results from a similar rat model also point to significant effects of extra virgin olive oil on H4K20m3 levels in mammary tumors or glands [77]. Tri-methylation of H3K4 has been shown to be highly regulated and tightly linked with active gene transcription, oncogenesis, cancer progression, and cancer-related functions [78]. In this regard, our results indicate the beneficial effects of *T. vulgaris* in mammary carcinogenesis.

Several pre-clinical and clinical BC studies have demonstrated that isolated phytochemicals or their mixtures modulate the expression of oncogenic or tumor-suppressive miRNAs. It is well known that RNA epigenetic mechanisms play an important regulatory role in the process of normal cell development and differentiation, as well as in the process of carcinogenesis [59]. Numerous miRNAs have been evaluated as BC risk predictors. MiR22, miR34a, miR210, and miR21 are well-documented non-coding RNAs in clinical and also preclinical BC research [79,80,81,82]. Our data on miRNA expressions in cancer cells in vivo have provided consistent results (with the exception of miR21 expression). The higher dose of *T. vulgaris* caused an unambiguous increase in miR22, miR34a, and miR210 expressions in rat mammary carcinomas. The two first RNAs are specific tumor-suppressive miRNAs, and miR210 behaves as a tumor-suppressive in the initial phase of oncogenesis characterized by normoxic conditions in tumor tissue [82,83]. The explanation for the dose-dependent biphasic effect of *T. vulgaris* on miR21 expression in BC cells in vivo needs further evaluation and comparison of the data within the oncological research; the answer to this phenomenon at this moment will just be speculation. We propose that positive significant changes in miRNAs expression contribute to the chemopreventive effects of *T. vulgaris* in rat mammary carcinogenesis.

Numerous cancer-related genes are subjects to methylation-dependent silencing. Hypermethylation of tumor-suppressor gene promoters plays a specific role in carcinogenesis [10]. Generally, the decrease in methylation status of gene promoters corresponds to an increase in the gene expression. Expression of tumor suppressor genes such as ATM, PITX2, RASSF1A, PTEN, and TIMP3 are frequently downregulated in BC patients [10] or in preclinical research [77]. In this regard, we have evaluated the methylation of specific CpG islands in the promoter regions of the above-mentioned tumor suppressor genes in rat mammary carcinomas in chemoprevention studies. In a previous study, strong chemopreventive effects of cloves in rats were accompanied with significant effects on the methylation levels of RASSF1A and TIMP3 promoters in different CpG islands [27]. Compared to clove buds as an excellent chemopreventive agent in rats, we found more unambiguous effects of *T. vulgaris* on the total methylation status, even across the four promoters, i.e., significant decreases in ATM, RASSF1A, PTEN, and TIMP3. However, results from our laboratory could be considered as one of the many mechanisms by which phytochemicals from *T. vulgaris* or clove buds may prevent chemically-induced rat mammary adenocarcinomas. Our results alluding to the epigenetic mechanism of action of *T. vulgaris* await further experimental and clinical validations. Despite extensive research focused on the human epigenome, there is still insufficient experimental data published on the epigenetic alterations induced by plant compounds. Moreover, most of the data are of preclinical nature that cannot be easily transferred to clinical practice. Future research needs to focus on phytochemicals which influence the human epigenome. More detailed insight is needed into the molecular and target mechanisms, determination of effective (individual) doses, the assessment of the combined administration of plant molecules targeting several relevant epigenetic mechanisms, the evaluation of compounds linked to cancer stem cell survival, and the individual characteristics concerning the personalized approach, or improving the bioavailability of phytochemicals in organisms [69].

Importantly, we note that the mechanisms of the anticancer action of *T. vulgaris* are model-dependent: in the rat model, significant changes in the parameters of angiogenesis, lipid-oxidation, CSCs, and epigenetics were noted, yet in the mouse model antiproliferative effects in 4T1 tumors were observed, and in the cancer cell lines proapoptotic and antiproliferative effects were recorded. BC is an accepted example of both phenotype and genomic heterogeneity of tumors. Cancer cells (from three different models) demonstrate different behavior according to genotype. Therefore, regarding the specific signaling pathway, some cancer cells (in vivo or in vitro) were more sensitive and others less sensitive to the effects of the mixture of phytochemicals from *T. vulgaris*. For this reason, *T. vulgaris* differentially targets specific signaling pathways according to the phenotype (genotype) of the cancer cells and also doses in rats, mice, and human cells. The connection between several BC models in our study provides a more complex picture and demonstrates the effectiveness of *T. vulgaris* against BC cells in vivo and in vitro.

The daily consumption of several different herbs and spices which are typical of the Mediterranean diet (strong antioxidants such as thyme, cloves, sumac, oregano, rosemary, and sage, etc.) appear to have more pronounced anti-cancer effects in humans compared to a single herb [7]. Innovative strategies which include individualized patient profiling [84], novel screening programs focused on the needs of young subpopulations [85], patient stratification utilizing specific phenotypes and genotypes known to be linked to increased prevalence of BC [86], and multiomic diagnostics are crucial clinical approaches to promoting a paradigm shift in the management of BC prediction, prevention, and personalized medicine [87,88,89].

## 4. Materials and Methods

The experiments were approved by the Ethical Commission of the Jessenius Faculty of Medicine of Comenius University (Protocol No. EK1860/2016) and by the State Veterinary and Food Administration of the Slovak Republic (Accreditation No. Ro-3239/15-221 and Ro-1640/17-221).

### 4.1. Animals and Induction of Mammary Carcinogenesis, and Design of Experiment

Sprague-Dawley female rats (Charles River Laboratories, Sulzfeld, Germany) and BALB/c female mice (Charles River Laboratories, Sulzfeld, Germany) at five weeks of age were used in the experiments. Animals were acclimatized to standard vivarium conditions, including temperature 23 ± 2 °C, relative humidity 40–60 %, and artificial regimen (L/D 12:12 h). During the experiment, rats and mice were fed with the Ssniff^®^ R-Z (M-Z) low-phytoestrogen V1354-0 diet (Soest, Germany) and had access to drinking water ad libitum. In rats, mammary carcinogenesis was induced by NMU (Sigma, Deisenhofen, Germany) administered intraperitoneally (a single dose of 50 mg/kg body weight on average on the 42nd postnatal day). This model mimics that seen in high-risk premenopausal women. In mice, a syngeneic model was used; mouse mammary adenocarcinoma 4T1 cells (1 × 10^5^ cells/mouse) were inoculated subcutaneously into the abdominal mammary gland area.

Chemoprevention with dried *T. vulgaris* (haulm, Calendula, Nová Ľubovňa, Slovak Republic; country of origin – Slovakia/Presov region) began one week before carcinogen administration and lasted until 15 weeks after NMU administration in rats. In mice, *T. vulgaris* administration was initiated from the day of 4T1 cell inoculation and lasted 15 days (until the end of the study). In both animal models, *T. vulgaris* was administered through the diet (haulm was cut into 2 mm pieces and processed via a “cold pelleting procedure”) at two concentrations of 1 g/kg (0.1%) and 10 g/kg (1%). Rats/mice (n = 25/26 per group, total 75/78 animals) were randomly assigned into three experimental groups: (1) the control group, which was without chemoprevention/treatment; (2) chemoprevention/treatment with *T. vulgaris* at a concentration of 0.1% (THYME 0.1); (3) chemoprevention/treatment with *T. vulgaris* at a concentration of 1% (THYME 1). The rats were weighed and palpated weekly in order to register the presence, number, location, and size of each palpable tumor. In mice, the growth of tumors was monitored (three times a week) from the third day after 4T1 cell inoculation and the size of palpated tumors for each mouse was recorded individually. Food intake over 24 hours was monitored four times in rats and twice in mice during the experiment. The average daily dose of *T. vulgaris* per rat was 16.27 mg (THYME 0.1) and 172.00 mg (THYME 1), and per mouse was 2.06 mg (THYME 0.1) and 15.13 mg (THYME 1). In the last week of the animal study, the animals were quickly decapitated, the blood from each animal was collected, mammary tumors were excised, and the tumor sizes were recorded.

### 4.2. Histopathological and Immunohistochemical Analysis of Rat and Mouse Tumors

A tissue sample of each rat and mouse adenocarcinoma was routinely formalin-fixed and paraffin-embedded. Rat tumors were classified according to the criteria for the standardized classification of mammary tumors. The additional parameter—grade of invasive carcinomas—was used. Rat tumor samples were divided into low-grade (LG) and high-grade (HG) carcinomas. The criteria for categorization (solidization, cell atypia, mitotic activity index, and necrosis) were chosen according to the standard diagnostic method of classification. HG carcinomas were considered to be tumors with ≥ 2 positive criteria; LG carcinomas were tumors with ≤ 1 positive criterion. In addition, the mitotic activity index and all tumor area/necrosis ratios were assessed in the mice tumors. Metabolic parameters (total cholesterol, very low-density lipoprotein cholesterol, low-density lipoprotein cholesterol, high-density lipoprotein cholesterol, triacylglycerols, and glucose) were evaluated in rat serum using an Olympus AU640 (Olympus Optical, Tokyo, Japan) automatic biochemical analyzer.

The most relevant part of rat mammary tumors in the paraffin block (which includes the typing characteristics and having the largest representation of vital tumor epithelial component, i.e., not having regressive changes such as extensive necrosis) was chosen for immunohistochemical analysis. The detection of selected markers for the mechanistic study was carried out by indirect immunohistochemical method on whole paraffin sections, utilizing commercially available rat–specific antibodies (Santa Cruz Biotechnology, Paso Robles, CA, USA; Dako, Glostrup, Denmark; Bioss, Woburn, MA, USA; GeneTex, Irvine, CA, USA; Abcam, Cambridge, MA, USA; Boster Biological Technology, Pleasanton, CA, USA; Thermo Fisher Scientific, Rockford, IL, USA). All steps of the immunohistochemical staining (Autostainer Link 48/Hermes/) were processed according to manufacturers’ recommendations, as described previously. The concentrations used for each primary antibody were: caspase-3 1:500, Bax 1:200, Bcl-2 1:200, Ki-67 1:50, VEGFA 1:150, VEGFR-2 1:80, MDA 1:1000, CD24 1:200, CD44 1:200, ALDH1A1 1:500, EpCam 1:160, H3K4m3 1:500, H3K9m3 1:400, H4K20m3 1:300, and H4K16ac 1:200. The primary antibodies were visualized by a secondary staining system (EnVision, Dual Link System-HRP, Cat. No. K060911, Dako North America, Carpinteria, CA, USA) using diaminobenzidine tetrahydrochloride as a substrate. Negative controls included the omission of primary antibodies. Immunohistochemically detected antigen expression was evaluated by precise morphometric method. Sections were screened and digital images at magnifications of ×400 were microscopically analyzed (Olympus BX41N, Tokyo, Japan). The expression of proteins was quantified as the average percentage of the antigen positive area in standard fields (0.5655 mm^2^) of tumor hot-spot areas. We analyzed three hot spots per tumor sample using the morphometric method. Morphometric analysis of the digital images was performed using QuickPHOTO MICRO software, version 3.0 (Promicra, Prague, Czech Republic). The values were compared between treated (THYME 0.1 and THYME 1) and non-treated (control) tumor cells of female rats; at least 60 tumor samples for one marker were analyzed (in total 900 tumor slides for 15 markers).

### 4.3. miRNA Expression Analysis

Total RNA was isolated from tumor tissues by using a commercially available preparation *miRVana microRNA Isolation Kit* (Thermo Fisher Scientific, Waltham, MA, USA). A detailed description of these procedures is available in the supplementary protocol. After the extraction, the RNA was quantified on a NanoDrop ND-2000 Spectrophotometer (Thermo Scientific, Wilmington, Delaware, USA). Reverse transcription was performed using the TaqMan Advanced miRNA cDNA Synthesis Kit (Applied Biosystems, Life Technologies, Carlsbad, CA, USA). The samples of cDNA were stored at −20 °C for future usage. For a quantitative real-time PCR, the miRNA-specific *TaqMan*™ *Advanced miRNA Assays Kit* (Applied Biosystems Life Technologies, Carlsbad, CA, USA) for tumor-suppressors miR-22, miR-210, miR-34a, and for the target oncogenic miR-21, was used. MiR-191-5p was selected as the internal control miRNA to normalize the cDNA levels of the samples. A quantitative real-time PCR reaction was performed on an AB7500 Real Time System (Applied Biosystems Life Technologies, Carlsbad, CA, USA). All qPCR reactions were performed in duplicate and Cq values were averaged.

### 4.4. Nucleic Acids Extraction and Bisulfite Conversion

Prior to the isolation of DNA, fresh frozen tumor samples were disrupted by TissueLyser LT (Qiagen, Hilden, Germany). An average of 50–100 mg of sample and stainless steel beads 5 mm in diameter (Qiagen, Germany) were added into the precooled tube. Samples were disrupted and homogenized in 200 μL of lysis buffer (Qiagen, Germany) in TissueLyser LT (Qiagen, Germany) at 50 Hz until the tissue was completely disturbed. Homogenized samples were incubated at 56 °C with addition of 20 μL of proteinase K. Genomic DNA was extracted using the DNeasy Blood and Tissue kit (Qiagen, Germany) according to the manufacturer’s protocol. DNA concentration was estimated by the Qubit™ 3.0 Fluorometer (Thermo Fisher Scientific) at a wavelength of 260 nm. At least 50 ng of DNA were used for sodium bisulfite modification using an EpiTect Bisulfite kit (Qiagen, Germany) according to the manufacturer’s recommendation.

### 4.5. Quantitative Methylation Analysis (Pyrosequencing)

Quantitative pyrosequencing was performed with the PyroMark PCR kit (Qiagen, Germany). Predesigned methylation assays were used to determine the methylation status of three CpG sites in the *RASSF1A*, six CpG islands in the *TIMP3*, six CpG islands in the *PTEN*, five CpG areas in the *PITX2,* and four CpG islands in the *ATM* promoter (PyroMark CpG assay, Qiagen, Germany). The total volume of the PCR reaction was 25 μL including 20 ng of bisulfite treated DNA. Thermal cycling protocol included an initial denaturation at 95 °C for 15 min, followed by 45 cycles of amplification: 94 °C for 30 s, 56 °C during 30 s and 72 °C for 30 s and a final extension at 72 °C for 10 min. The amplification products were confirmed by electrophoresis on 1.75% agarose gel, stained with GelRed Nucleic Acid (Biotinum Inc., Fremont, CA, USA), and visualized on a UV transilluminator. Obtained PCR products were analyzed according to the manufacturer’s instructions using the PyroMark Q96 ID System (Qiagen, Germany) with PyroMark Gold Q96 Reagents. Methylation data were evaluated with the instrument software (PyroMark Q96 software version 2.5.8; Qiagen, Germany).

### 4.6. Cell Culture and Experimental Design

The human cancer cell line MCF-7 (human breast adenocarcinoma; ER+, PR+, HER2-) and MDA-MB-231 (human breast adenocarcinoma; ER-, PR-, HER2-) were cultured in Dulbecco’s modified Eagle’s medium with Glutamax-I and Sodium pyruvate (GE Healthcare, Piscataway, NJ, USA) and RPM1 1640 medium (Biosera, Kansas City, MO, USA), respectively. The growth medium was supplemented with 10% fetal bovine serum (Gibco, Thermo Fisher Scientific and 1X HyClone™ Antibiotic/Antimycotic Solution (GE Healthcare), and cells were cultivated in an atmosphere containing 5% CO_2_ in humidified air at 37 °C. Cell viability, estimated by trypan blue exclusion, was greater than 95% before each experiment.

MCF-7 (3 × 10^5^) and MDA-MB-231 (1 × 10^5^) cells were seeded in Petri dishes and cultivated for 24 h in a complete medium with 10% FCS. Cells were treated with essential oil of *T. vulgaris* (EOT, Calendula, Nová Ľubovňa, Slovak Republic) for 24, 48, and 72 h prior to analysis. EOT was prepared by a steam distillation procedure. Its composition was analyzed by GC-MS, as is described later.

### 4.7. Cytotoxicity Assay

The MTS colorimetric assay was used to determine the cytotoxic effects of EOT at final dilutions of 0.72–0.023 µg/ml (MCF-7) or 0.72–0.0056 µg/ml (MDA-MB-231). After 72 h of incubation, 10 µL of MTS (Promega, Madison, WI, USA) was added to each well according to the CellTiter 96^®^ AQueous One Solution Cell Proliferation Assay protocol. After a minimum 1 h of incubation, the absorbance was measured at 490 nm using the automated Cytation^TM^ 3 Cell Imaging Multi-Mode Reader (Biotek, Winooski, VT, USA). The absorbance of the control wells was taken as 100% and the results were expressed as a fold of the control. All experiments were performed in triplicate.

### 4.8. 5-Bromo-20-deoxyuridine (BrdU) Cell Proliferation Assay

BrdU incorporation was used to analyze cell proliferation activity and was monitored by quantification of BrdU introduced to the genomic DNA during cell growth after EOT treatment (final dilutions ranged between 0.72–0.023 µg/mL and 0.72–0.0056 µg/mL, respectively). DNA synthesis was assessed using a colorimetric cell proliferation ELISA assay (Roche Diagnostics GmbH, Mannheim, Germany) following the manufacturer’s protocol. The color intensity was measured with a Cytation^TM^ 3 Cell Imaging Multi-Mode Reader (Biotek, Winooski, VT, USA at 450 nm (reference wavelength: 690 nm). The results were expressed as a fold of the control. All experiments were performed in triplicate. For the following analyses final dilutions (calculated from MTS and BrdU assays) of 0.12 µg/mL (MDA-MB-231 cells) or 0.13 µg/mL (MCF-7 cells) were used.

### 4.9. Flow Cytometry Analyses Protocol

For flow cytometric analysis (FCM), floating and adherent cells were harvested together at 24, 48, and 72 h after treatment (EOT final dilutions were 0.12 or 0.13 µg/mL), washed in PBS, re-suspended in PBS, and stained prior to analysis. Fluorescence was detected, and after 15–30 min of incubation in the dark at room temperature, a FACSCalibur flow cytometer (Becton Dickinson, San Jose, CA, USA) was used.

Staining solutions used within in vitro analyses are summarized in Table 8.

### 4.10. Examination of Plant Secondary Metabolites in Essential Oil of T. vulgaris

Semi-quantitative analysis using GC-MS was used for the evaluation of the phytochemical profile of *T. vulgaris* essential oil. An Agilent Technologies GC 7890A system (Santa Clara, CA, USA) equipped with a DB-WAXetr column (60 m × 320 µm × 0.25 µm film thickness, Santa Clara, CA, USA) with a 5975C VL MSED and Triple-Axis detector (Santa Clara, CA, USA) was used. The method settings were as follows: injector 250 °C, injection volume 0.1 µL, pressure 72.313 kPa; oven program 40 °C for 5 min, then 4 °C/min to 250 °C for 2.5 min, run time 60 min, and inlet gas He with average velocity 34.4 cm/s. The MS detector settings were: MS source 230 °C, MS Quad 150 °C, solvent delay time 6 min, MS Scan 29–550 *m/z*.

### 4.11. Statistical Analyses

In the in vivo study, data were expressed as mean ± SEM. The Mann-Whitney test, Kruskal-Wallis test, Student’s t-test, and one-way analysis of variance (ANOVA) were the statistical methods used in data evaluation. Tumor volume was calculated according to the formula: V = π × (S_1_)^2^ × S_2_/12 (S_1_, S_2_ are tumor diameters; S_1_ < S_2_). In the fluorescence assay, ANOVA was first carried out to test the differences between groups; comparisons between individual groups were made using a Student-Newman-Keuls multiple comparisons test. In the in vitro study, data were expressed as mean ± SD. Data were analyzed using ANOVA followed by a Bonferroni multiple comparisons test. Differences were considered significant when *p* < 0.05. The quantitative results were calculated from calibration curves, expressed as mean ± SD. Data analyses were conducted using GraphPad Prism, version 5.01 (GraphPad Software, La Jolla, CA, USA). The examinations of plant secondary metabolites in the essential oil of *T. vulgaris* were performed in triplicate.

## 5. Conclusions

Dietary habits and phytochemicals are of particular interest for effective cancer prevention and targeted therapy. However, clinical research has not sufficiently supported the concept that whole plant foods may decrease cancer risks. This study has, for the first time, demonstrated significant anti-cancer activity of *T. vulgaris* (haulm) in experimental mammary carcinogenesis using two in vivo models (chemoprevention and therapeutic) and two BC cell lines in vitro. The results have demonstrated several mechanisms of anticancer action against experimental BC (proapoptotic, antiproliferative, antiangiogenic, antioxidative, and anti-CSCs effects). Specifically, the significant effect of *T. vulgaris* on several epigenetic mechanisms in mammary carcinogenesis warrants further attention in oncological research. Results from our laboratory suggest that regular consumption of *T. vulgaris* could reduce the risk of BC in humans. However, determining the dosage and adverse events of the long-term administration of *T. vulgaris* in humans will require clinical evaluation.

## Figures and Tables

**Table 1:** Effects of *T. vulgaris* in *N*-methyl-*N*-nitrosourea-induced mammary carcinogenesis in female Sprague-Dawley rats at the end of experiments.

Group	CONT	THYME 0.1	THYME 1
Tumor bearing/all animals	16/24	17/25	13/25
Tumor frequency per group *	1.54 ± 0.38	2.00 ± 0.43 (+30%)	0.72 ± 0.19 ^a,b^ (−53%)
Tumor latency * (days)	80.29 ± 5.34	88.28 ± 3.89 (+8 days)	86.15 ± 6.65 (+6 days)
Tumor incidence (%)	66.7	68.0 (+2%)	52.0 (−22%)
Average tumor volume * (cm^3^)	0.46 ± 0.14	0.36 ± 0.09 (−21.5%)	0.52 ± 0.16 (+13%)
Cumulative tumor volume ** (cm^3^)	15.81	18.19 (+15%)	8.90 (−43.5%)

CONT—control group, THYME 0.1—group with administered *T. vulgaris* at a concentration of 1 g/kg in diet, THYME 1—group with administered *T. vulgaris* at a concentration of 10 g/kg in diet. * Data are expressed as mean ± SEM. ** Data are expressed as a sum of volumes per group. Values in brackets are calculated as %-ual deviation from the 100% of non-influenced control group (with the exception of latency). Significantly different, ^a^ *p* < 0.01 versus CONT; ^b^ *p* < 0.01 versus THYME 0.1.

**Table 2:** Histopathological characteristics of 4T1 tumors in Balb/c mice after *T. vulgaris* treatment.

Parameter	CONT	THYME 0.1	THYME 1
**Necrosis/all tumor area**	8.71 ± 3.08	1.99 ± 1.11 **	1.66 ± 1.16 **
**Mitotic activity index**	25.15 ± 2.32	17.26 ± 1.82 *	18.82 ± 1.99 *

Data are expressed as mean ± SEM. Significant difference: * *p* < 0.05, ** *p* < 0.01 versus CONT.

**Table 3:** The cell cycle distribution in MCF-7 cells after thyme essential oil (EOT) treatment.

Time (h)	24		48		72	
Treatment	CONT	EOT	CONT	EOT	CONT	EOT
**Sub-G_0_/G_1_**	1.59 ± 0.41	10.86 ± 0.36 *	1.73 ± 0.41	19.69 ± 1.38 **	2.93 ± 0.43	23.51 ± 2.75 **
**G_1_**	70.47 ± 1.55	62.53 ± 2.54 *	68.83 ± 3.73	47.80 ± 3.24 **	70.53 ± 0.44	50.20 ± 3.41 **
**S**	14.90 ± 1.41	13.83 ± 0.28	15.60 ± 1.62	18.25 ± 1.35	13.50 ± 0.78	12.87 ± 1.02
**G_2_/M**	13.03 ± 2.34	12.77 ± 0.27	13.83 ± 1.00	14.25 ± 0.43	13.03 ± 2.18	13.39 ± 0.41

The cell cycle distribution in MCF-7 cells after EOT treatment (0.12 µg/mL) was assessed by flow cytometry. Data are expressed as the mean ± standard deviation (SD) of three independent experiments. Significant differences between control and EOT-treated cells are shown: * *p* < 0.05, ** *p* < 0.01.

**Table 4:** Cell cycle distribution in MDA-MB-231 cells after EOT treatment.

Time (h)	24		48		72	
Treatment	CONT	EOT	CONT	EOT	CONT	EOT
**Sub-G_0_/G_1_**	1.13 ± 0.20	2.46 ± 0.43	0.54 ± 0.12	3.13 ± 0.33 *	0.70 ± 0.11	7.47 ± 0.55 *
**G_1_**	51.60 ± 2.56	60.70 ± 0.90 *	58.80 ± 0.33	60.67 ± 3.26	64.83 ± 1.84	58.25 ± 2.21
**S**	21.55 ± 1.38	14.10 ± 2.40 *	19.80 ± 0.85	17.57 ± 1.19	15.27 ± 0.78	17.75 ± 2.07
**G_2_/M**	25.75 ± 1.13	22.75 ± 3.75	20.87 ± 1.09	18.63 ± 1.15	19.20 ± 2.25	16.55 ± 1.03

Cell cycle distribution in MDA-MB-231 cells after EOT treatment (0.13 µg/mL) was assessed by flow cytometry. Data are expressed as the mean ± SD of three independent experiments. Significant differences between control and EOT-treated cells are shown: * *p* < 0.05.

**Table 5:** Induction of apoptosis in MCF-7 cells after EOT treatment.

Time (h)	24		48		72	
Treatment	CONT	EOT	CONT	EOT	CONT	EOT
**An** ^−^ **/PI** ^−^	86.67 ± 2.25	61.75 ± 0.42 **	71.07 ± 3.01	46.90 ± 3.76 **	88.73 ± 3.47	32.47 ± 3.90 ***
**An^+^/PI** ^−^	4.64 ± 0.72	29.86 ± 3.75 **	8.23 ± 1.20	41.13 ± 4.45 ***	3.57 ± 0.57	39.97 ± 2.20 ***
**An^+^/PI^+^**	3.00 ± 0.53	3.59 ± 0.88	10.27 ± 0.87	7.75 ± 0.73	2.53 ± 1.10	11.71 ± 2.21 *
**An** ^−^ **/PI^+^**	5.69 ± 0.41	4.79 ± 1.24	10.43 ± 1.12	4.21 ± 1.05	5.17 ± 1.06	15.84 ± 0.74 *

The induction of apoptosis in MCF-7 cells after EOT treatment (0.12 µg/mL) was analyzed after annexin V and PI staining protocol for flow cytometry. The percentage of events in the non-apoptotic (lower left, An^−^/PI^−^), early apoptotic (lower right, An^+^/PI^−^), late apoptotic (upper right An^+^/PI^+^) and necrotic (upper left, An^−^/PI^+^) quadrants is indicated. Values are the mean ± SD of three independent experiments. Significant differences between control and EOT-treated cells are shown: * *p* < 0.05, ** *p* < 0.01, *** *p* < 0.001.

**Table 6:** Induction of apoptosis in MDA-MB-231 cells after EOT treatment.

Time (h)	24		48		72	
Treatment	CONT	EOT	CONT	EOT	CONT	EOT
**An** ^−^ **/PI** ^−^	88.07 ± 1.43	71.97 ± 2.62 *	92.27 ± 1.40	72.60 ± 3.05 **	93.47 ± 2.21	62.47 ± 1.70 **
**An^+^/PI** ^−^	5.78 ± 0.93	17.15 ± 0.91 *	1.58 ± 0.44	19.40 ± 0.25 **	1.02 ± 0.12	27.15 ± 1.40 **
**An^+^/PI^+^**	2.06 ± 0.33	4.06 ± 0.37	2.18 ± 0.06	4.34 ± 0.26	1.38 ± 0.17	5.35 ± 0.36 *
**An** ^−^ **/PI^+^**	4.12 ± 0.29	6.81 ± 1.77	3.98 ± 0.24	3.66 ± 0.67	4.13 ± 0.07	5.06 ± 0.85

The induction of apoptosis in MDA-MB-231 cells after EOT treatment (0.13 µg/mL) was analyzed after annexin V and PI staining protocol for flow cytometry. The percentage of events in the non-apoptotic (lower left, An^−^/PI^−^), early apoptotic (lower right, An^+^/PI^−^), late apoptotic (upper right An^+^/PI^+^) and necrotic (upper left, An^−^/PI^+^) quadrants is indicated. Values are the mean ± SD of three independent experiments. Significant differences between control and EOT-treated cells are shown: * *p* < 0.05, ** *p* < 0.01.

**Table 7:** Relative abundance of major compounds of *T. vulgaris* essential oil.

No.	Compound	Retention Time [min]	Relative Content [%]
**1**	**α-Pinene**	9.423	2.855
**2**	**Camphene**	10.919	0.748
**3**	**β-Pinene**	12.424	0.579
**4**	**β-Myrcene**	14.752	1.035
**5**	**Limonene**	15.957	0.742
**6**	**Eucalyptol**	16.262	0.694
**7**	***p*-Cymene**	18.875	43.108
**8**	**Linalool**	28.072	4.555
**9**	**β-Caryophyllene**	29.682	0.947
**10**	**α-Terpineol**	32.561	1.066
**11**	**Borneol**	32.695	0.745
**12**	**Thymol**	45.154	39.769
**13**	**Carvacrol**	45.787	0.681
	**Total**	-	97.524

Only compounds exceeding 0.5% of relative content are shown.

**Table 8:** Staining solutions used within in vitro analyses.

Analyses	Staining Solution	Company
Cell cycle *	10% Triton X-100 0.5 mg/ml ribonuclease A 0.025 mg/ml propidium iodide (PI) In 500 µL PBS	Sigma-Aldrich, Steinheim, Germany
**Apoptosis**	Annexin V-Alexa Fluor 647 1:100	Thermo Scientific, Rockford, IL, USA
	PI (5mg/ml) 1:500	Sigma-Aldrich
**Mitochondrial membrane potential**	TMRE (tetramethylrhodamine ethyl ester per chlorate) final conc. 0.1 µM	Molecular Probes, Eugene, OR, USA
**Caspase activation**	Cleaved Caspase-3 rabbit monoclonal antibody (mAb) phycoerythrin (PE) mAb PE conjugate 1:100	Cell Signaling, Danvers, MA, USA
	Cleaved Caspase-7 rabbit mAb PE conjugate 1:100	
**Protein analysis**	Cleaved PARP rabbit mAb PE conjugate 1:100	
	Bcl-2 mouse mAb PE conjugated 1:100	
	Phospho-Bcl-2 (Ser 70) rabbit mAb Alexa Fluor 488 conjugate 1:200	

* After harvesting, cell suspension was fixed in cold 70% ethanol and kept at −20 °C overnight.

## Supplementary Materials

Supplementary materials can be found at https://www.mdpi.com/1422-0067/20/7/1749/s1.
