# Supplementary material for: Anticancer Activities of Thymus vulgaris L. in Experimental Breast Carcinoma In Vivo and In Vitro
Source: Int J Mol Sci. 2019 Apr 9;20(7):1749. doi: 10.3390/ijms20071749 (PMC6479806; doi:10.3390/ijms20071749)
Supplement: Supplementary file 1 [file ijms-20-01749-s001.pdf]

PROTOCOL

ambion® | RNA
by life technologies™

*mir*Vana™ miRNA Isolation Kit

life technologies™

For Research Use Only. Not intended for any animal or human therapeutic or diagnostic use.

Information in this document is subject to change without notice.

APPLIED BIOSYSTEMS DISCLAIMS ALL WARRANTIES WITH RESPECT TO THIS DOCUMENT, EXPRESSED OR IMPLIED, INCLUDING BUT NOT LIMITED TO THOSE OF MERCHANTABILITY OR FITNESS FOR A PARTICULAR PURPOSE. TO THE FULLEST EXTENT ALLOWED BY LAW, IN NO EVENT SHALL APPLIED BIOSYSTEMS BE LIABLE, WHETHER IN CONTRACT, TORT, WARRANTY, OR UNDER ANY STATUTE OR ON ANY OTHER BASIS FOR SPECIAL, INCIDENTAL, INDIRECT, PUNITIVE, MULTIPLE OR CONSEQUENTIAL DAMAGES IN CONNECTION WITH OR ARISING FROM THIS DOCUMENT, INCLUDING BUT NOT LIMITED TO THE USE THEREOF, WHETHER OR NOT FORESEEABLE AND WHETHER OR NOT APPLIED BIOSYSTEMS IS ADVISED OF THE POSSIBILITY OF SUCH DAMAGES.

LIMITED USE LABEL LICENSE: RESEARCH USE ONLY
The purchase of this product conveys to the purchaser the limited, non-transferable right to use the purchased amount of the product only to perform internal research for the sole benefit of the purchaser. No right to resell this product or any of its components is conveyed expressly, by implication, or by estoppel. This product is for internal research purposes only and is not for use in commercial applications of any kind, including, without limitation, quality control and commercial services such as reporting the results of purchaser's activities for a fee or other form of consideration. For information on obtaining additional rights, please contact **outlicensing@lifetech.com** or Out Licensing, Life Technologies, 5791 Van Allen Way, Carlsbad, California 92008.

TRADEMARKS

The trademarks mentioned herein are the property of Life Technologies Corporation or their respective owners.

© 2011 Life Technologies Corporation. All rights reserved.

Part Number 1560M Rev. C
01/2011

*mir*Vana™ miRNA Isolation Kit

Part Numbers AM1560, AM1561

Protocol

I. Introduction . . . 1

A. Background

B. Summary of the *mir*Vana miRNA Isolation Kit Procedure

C. Reagents Provided with the Kit and Storage

D. Materials Not Provided with the Kit

E. Related Products

II. *mir*Vana miRNA Isolation Kit Procedure . . . 7

A. Prepare the Wash Solutions

B. Equipment Preparation

C. Sample Type and Amount

D. Cell Lysis and Tissue Disruption

E. Organic Extraction

F. Final RNA Isolation

G. Analyzing RNA after Purification

III. Troubleshooting . . . 16

A. RNA Appears Degraded on Gel

B. RNA Yield Is Lower than Expected or Inconsistent

C. Contaminants in RNA; RNA Inhibits Enzymatic Reactions

IV. Additional Procedures . . . 19

A. Isolation of Small RNAs from Total RNA Samples

B. Assessing RNA Concentration and Purity by UV Absorbance

C. Gel Analysis of Small RNAs

D. Detection of Small RNAs by Northern Blot

V. Appendix . . . 26

A. References

B. Quality Control

C. Safety Information

VI. Documentation and Support . . . 29

A. Obtaining SDSs

B. Obtaining support

I. Introduction

IMPORTANT

Before using this product, read and understand the "Safety Information" in the appendix in this document.

A. Background

In the past few years, interest in the identification, detection, and use of small RNA molecules has rapidly expanded. This interest is the result of two related lines of research. In one, small double-stranded RNAs (dsRNAs) called small interfering RNAs (siRNAs) are used to silence the expression of specific genes at the post-transcriptional level by a pathway known as RNA interference (RNAi). In the other, numerous small regulatory RNA molecules, referred to as microRNAs (miRNAs), have been shown to regulate target gene expression in various organisms. Both miRNAs and siRNAs range between 15–30 nucleotides in length and are biologically significant. Evidence is accumulating that these molecules are related, and share common processing pathways (Zeng 2003).

The *mir*Vana™ miRNA Isolation Kit was designed for purification of RNA suitable for studies of both siRNA and miRNA in natural populations. The kit employs an organic extraction followed by immobilization of RNA on glass-fiber filters to purify either total RNA, or RNA enriched for small species, from cells or tissue samples.

B. Summary of the *mir*Vana miRNA Isolation Kit Procedure

Traditional RNA isolation methods are not well suited for isolation of small RNAs

Variations of two methods have historically been used to prepare RNA from natural sources (e.g. tissue samples, whole organisms, cell cultures, bodily fluids): chemical extraction and immobilization on glass, often referred to as solid-phase extraction. Chemical extraction methods usually use highly concentrated chaotropic salts in conjunction with acidic phenol or phenol-chloroform solutions to inactivate RNases and purify RNA from other biomolecules. These methods provide very pure preparations of RNA; however, the RNA must typically be desalted and concentrated with an alcohol precipitation step. Routine alcohol precipitation does not quantitatively recover small nucleic acid molecules, making it ill-suited for the preparation of very small RNAs.

The second method, solid-phase extraction, relies on high salt or salt and alcohol to decrease the affinity of RNA for water and increase its affinity for the solid support used. The use of glass (silica) as a solid support has been shown to work for large RNAs in the presence of high

concentrations of denaturing salts (Boom, et al. 1990). The conditions routinely used for solid-phase purification of RNA, however, do not effectively recover small RNAs.

Isolate total RNA or RNA that is highly enriched for small RNAs

The *mir*Vana miRNA Isolation procedure combines the advantages of organic extraction and solid-phase extraction, while avoiding the disadvantages of both. High yields of ultra-pure, high quality, small RNA molecules can be prepared in about 30 min (Figure 1).

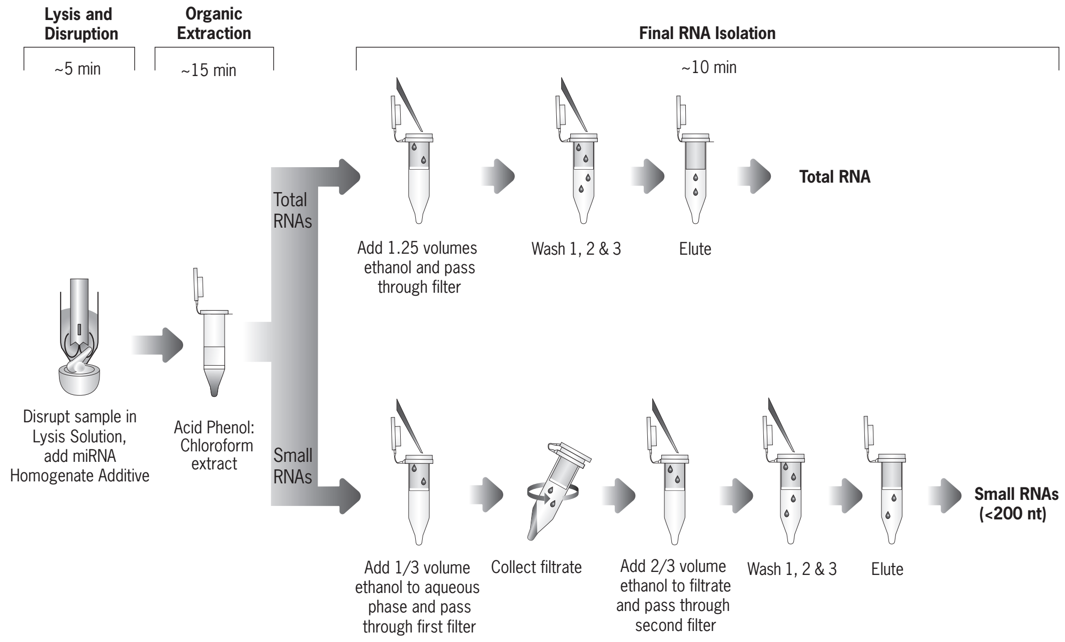

Figure 1. Overview of the *mir*Vana™ miRNA Isolation Kit Procedure.

The sample is first lysed in a denaturing lysis solution which stabilizes RNA and inactivates RNases. The lysate is then extracted once with Acid-Phenol:Chloroform which removes most of the other cellular components, leaving a semi-pure RNA sample. This is further purified over a glass-fiber filter by one of two procedures to yield either total RNA or a size fraction enriched in miRNAs. The glass-fiber filter procedure uses solutions formulated specifically for miRNA retention to avoid the loss of small RNAs that is typically seen with glass-fiber filter methods.

Sample disruption and organic extraction

The first step of the *mir*Vana miRNA Isolation Kit procedure is to disrupt samples in a denaturing lysis buffer. Next, samples are subjected to Acid-Phenol:Chloroform extraction which provides a robust front-end purification that also removes most DNA (Chomczynski, 1987).

Final RNA purification over glass-fiber filter

At this point there are separate procedures for purification of either total RNA—including very small RNA species—or for purifying RNA that is highly enriched for small RNA species and contains very little RNA larger than about 200 bases.

IMPORTANT

To isolate RNA for miRNA expression profiling using miRNA arrays, we recommend following the procedure for total RNA isolation (not the enrichment procedure for small RNAs). This makes it possible to both quantitate the RNA and to critically evaluate its quality, to verify that it is suitable for array analysis. Then further purify the miRNA population (e.g., with the flashPAGE™ system) before labeling samples for miRNA array analysis.

Final purification of total RNA

The procedure for isolation of total RNA is similar to routine glass-fiber binding procedures. Ethanol is added to samples, and they are passed through a Filter Cartridge containing a glass-fiber filter which immobilizes the RNA. The filter is then washed a few times, and finally the RNA is eluted with a low ionic-strength solution. Figures 2 and 3 on page 3 compare RNA isolated using the *mir*Vana miRNA Isolation Kit to RNA isolated using more traditional techniques.

Final purification of RNA enriched for small RNAs

To isolate RNA that is highly enriched for small RNA species, 100% ethanol is added to bring the samples to 25% ethanol. When this lysate/ethanol mixture is passed through a glass-fiber filter, large RNAs are immobilized, and the small RNA species are collected in the filtrate. The ethanol concentration of the filtrate is then increased to 55%, and it is passed through a second glass-fiber filter where the small RNAs become immobilized. This RNA is washed a few times, and eluted in a low ionic strength solution. Using this novel approach consisting of two sequential filtrations with different ethanol concentrations, an RNA fraction highly enriched in RNA species ≤200 nt can be obtained.

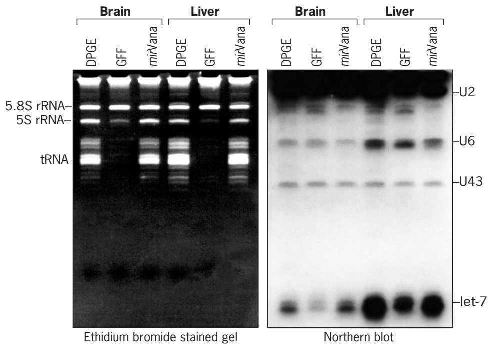

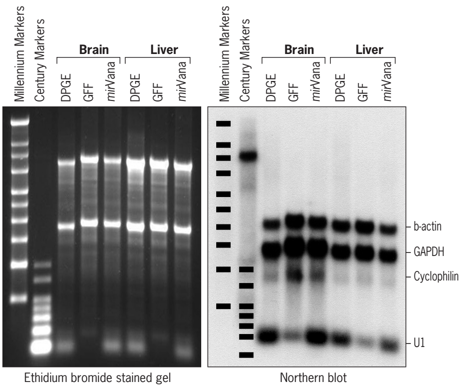

Figure 2. Comparison of miRNA Preps on Acrylamide Gel and Northern Blot.

miRNA was prepared from two different tissues by double phenol/guanidinium extraction (DPGE), glass-fiber filter (GFF), and *mir*Vana miRNA Isolation Kit. 2 µg of sample was compared on 15% denaturing polyacrylamide gel, stained with ethidium bromide and, in a Northern blot, hybridized with probes for the RNAs indicated.

Figure 3. Comparison of Total RNA Preps on Agarose Gel and Northern Blot.

Total RNA was prepared from two different tissues by DPGE, GFF, and *mir*Vana miRNA Isolation Kit. 1 µg of RNA obtained was run on 1% denaturing agarose gel, stained with ethidium bromide and, in a Northern blot, hybridized with probes for the RNAs indicated.

C. Reagents Provided with the Kit and Storage

This kit contains reagents for 40 isolations of total RNA, or 20 isolations of small RNAs.

To help address customer concerns about the costs of shipping organic chemicals such as Acid-Phenol:Chloroform, the *mir*Vana miRNA Isolation Kit is available both with and without Acid-Phenol:Chloroform, which is required for the procedure. Acid-Phenol:Chloroform (100 mL), P/N AM9720 is available separately.

P/N AM1560	P/N AM1561	Component	Storage
30 mL	30 mL	miRNA Wash Solution 1<br>Add 21 mL 100% ethanol before use	room temp*
50 mL	50 mL	Wash Solution 2/3<br>Add 40 mL 100% ethanol before use	room temp*
80	80	Collection Tubes	room temp
40	40	Filter Cartridges	room temp
100 mL	100 mL	Lysis/Binding Buffer	4°C
10 mL	10 mL	miRNA Homogenate Additive	4°C
100 mL	— †	Acid-Phenol:Chloroform	4°C
1.4 mL	1.4 mL	Gel Loading Buffer II	–20°C
5 mL	5 mL	Elution Solution	any temp‡

* Store at room temp for up to 1 month. For longer term storage, store at 4°C, but warm to room temp before use.

† Acid-Phenol:Chloroform is not supplied with P/N AM1561, but is required for the procedure. It is available as P/N AM9720.

‡ Store Elution Solution at –20°C, 4°C or room temp

Note that the kit is shipped at room temperature which will not affect its stability.

D. Materials Not Provided with the Kit

- If you purchased P/N AM1561, the *mir*Vana miRNA Isolation Kit that is supplied ***without*** Acid-Phenol:Chloroform, you will need to purchase P/N AM9720 Acid-Phenol:Chloroform (100 mL) in order to follow the procedure.
- RNase-free 1.5 mL or 0.5 mL polypropylene microfuge tubes, adjustable pipettors and RNase-free tips
  Note that Eppendorf® PhaseLock Gel™ is ***not*** compatible with the *mir*Vana miRNA Isolation Kit.
- ACS grade 100% ethanol

- Microcentrifuge capable of at least 10,000 x g
- (optional) Vacuum manifold: to pull solutions through the Filter Cartridges
- Apparatus and reagents for preparing and running denaturing acrylamide gels (see section IV.C. Gel Analysis of Small RNAs starting on page 22)
- (optional) Plant RNA Isolation Aid to remove polysaccharides and polyphenolics from plant tissues.

E. Related Products

RNase*Zap*® Solution<br>P/N AM9780, AM9782, AM9784	RNase*Zap* RNase Decontamination Solution is simply sprayed, poured, or wiped onto surfaces to instantly inactivate RNases. Rinsing twice with distilled water will eliminate all traces of RNase and RNase*Zap* Solution.
RNA*later*®-ICE Solution<br>P/N AM7030, AM7031	RNA*later*-ICE Frozen Tissue Transition Solution is designed to make it easier to process frozen tissue samples for RNA isolation. Simply drop frozen tissues into RNA*later*-ICE Solution and store overnight at –20°C. Once tissues are treated they can be easily processed using standard RNA isolation procedures.
RNA*later*® Solution<br>P/N AM7020, AM7021	RNA*later* Tissue Collection: RNA Stabilization Solution is an aqueous sample collection solution that stabilizes and protects cellular RNA in intact, unfrozen tissue and cell samples. RNA*later* Solution eliminates the need to immediately process samples or to freeze samples in liquid nitrogen. Samples can be submerged in RNA*later* Solution for storage at RT, 4°C, or –20°C without jeopardizing the quality or quantity of RNA that can be obtained.
flashPAGE™ Fractionator<br>P/N AM13100	The flashPAGE Fractionator is a specialized electrophoresis instrument for rapid PAGE-purification of small nucleic acids. Designed for use with flashPAGE Pre-Cast Gels and the optimized running buffers supplied in the flashPAGE Buffer Kit, the flashPAGE Fractionator purifies small nucleic acid molecules more quickly, easily, and efficiently than traditional PAGE purification.
*mir*Vana™ miRNA Bioarrays<br>see our web or print catalog	*mir*Vana miRNA Bioarrays are high-quality microarrays preprinted with probes representing a comprehensive panel of currently known human, mouse, and rat microRNAs (miRNAs) using GE Healthcare's CodeLink 3-D Gel Matrix slide surface for maximum interaction between probe and target. *mir*Vana miRNA Bioarrays are designed for analysis of miRNA samples labeled with the *mir*Vana miRNA Labeling Kit, using the positive control, hybridization, and wash components supplied with the *mir*Vana miRNA Bioarray Essentials Kit (P/N AM1567).
*mir*Vana™ qRT-PCR Primer Sets and qRT-PCR miRNA Detection Kit<br>P/N AM1558 and<br>see our web or print catalog	The *mir*Vana qRT-PCR Primer Sets and qRT-PCR miRNA Detection Kit provide a novel detection system for specific small RNAs. Each qRT-PCR Primer Set includes a primer for reverse transcription and a PCR primer pair optimized for sensitive detection of specific miRNAs by qRT-PCR. *mir*Vana qRT-PCR Primer Sets are available for a comprehensive selection of the human, mouse, and rat miRNAs in the miRNA Registry, as well as for exclusive ambi-miRs. The qRT-PCR miRNA Detection Kit provides reagents for amplification of the miRNA targeted by the qRT-PCR Primer Set via traditional endpoint RT-PCR or real-time RT-PCR using SYBR® Green I, and a control primer set.

*mir*Vana™ miRNA Detection Kit<br>P/N AM1552	The *mir*Vana miRNA Detection Kit provides an extremely sensitive solution hybridization assay capable of detecting attomole amounts of RNA. In addition, it can be used to simultaneously detect several small RNAs such as miRNA and siRNA, or both small RNA and long RNA species in the same sample. For a complete solution for small RNA analysis, use this kit in conjunction with the *mir*Vana miRNA Probe Construction Kit and/or the *mir*Vana Probe & Marker Kit.
Decade™ Markers<br>P/N AM7778	The Decade Marker System is a set of reagents to prepare radiolabeled low molecular weight RNA markers: from 10–100 nt in 10 nt increments. The user supplies only [γ-^{32}P]ATP to end label a single, gel purified RNA transcript which is then cleaved into the 10 molecular weight markers in a simple 5 minute reaction.
PAGE Reagents<br>see our web or print catalog	We offer high-quality reagents for polyacrylamide gel electrophoresis (PAGE), including acrylamide solutions for RNA, DNA and protein gels, ultra-pure molecular biology grade urea, and premixed acrylamide/urea solutions for simple preparation of denaturing gels.
NorthernMax® Kits<br>P/N AM1940, AM1946	The NorthernMax Kits: NorthernMax, and NorthernMax-Gly, combine ultrasensitive, reliable Northern blot protocols with unsurpassed quality control to ensure optimal results in less time.

II. *mir*Vana miRNA Isolation Kit Procedure

A. Prepare the Wash Solutions

NOTE

Once ethanol has been added, cap the Wash Solution bottles tightly to prevent evaporation.

Add 21 mL ethanol to miRNA Wash Solution 1

Add 21 mL of ACS grade 100% ethanol to the bottle labeled miRNA Wash Soln 1. Mix well. Place a check mark in the empty box on the label to indicate that the ethanol has been added.

Add 40 mL ethanol to Wash Solution 2/3

Add 40 mL of ACS grade 100% ethanol to the bottle labeled Wash Solution 2/3. Mix well. Place a check mark in the empty box on the label to indicate that the ethanol has been added.

NOTE

A precipitate may form in the Wash Solution 2/3 bottle over the next several days as excess EDTA falls out of solution. Simply leave these crystals in the bottle when removing Wash Solution for use.

B. Equipment Preparation

a. **Lab bench and pipettors**
Before working with RNA, it is always a good idea to clean the lab bench, and pipettors with an RNase decontamination solution (for example, Ambion RNase*Zap*® Solution).

b. **Gloves and RNase-free technique**
Wear laboratory gloves at all times during this procedure and change them frequently. They will protect you from the reagents, and they will protect the RNA from nucleases that are present on your skin.Use RNase-free pipette tips to handle the wash solutions and the Elution Solution, and avoid putting used tips into the kit reagents.

c. **Preparing equipment**
The equipment used for tissue disruption/homogenization should be washed well with detergent and rinsed thoroughly. Baking is unnecessary, because the Lysis/Binding Solution will inactivate most RNase contamination. If samples are to be ground in a mortar and pestle, prechill the equipment in dry ice or liquid nitrogen.

C. Sample Type and Amount

Sample type

This procedure is designed for small scale RNA isolation from plant and animal tissue or cultured cells, bacteria, yeast, viral particles, or enzyme reactions. It can be used with fresh or frozen cultured mammalian cells, or cells stored in RNA*later* or RNA*later*-ICE. It can also be used with fresh, frozen, or RNA*later*-stored animal tissues. This kit is compatible with Ambion Plant RNA Isolation Aid (P/N AM9690).

The *mir*Vana miRNA Isolation Kit can also be used to enrich total RNA samples for the small RNA fraction. These instructions are provided in section *IV.A. Isolation of Small RNAs from Total RNA Samples* on page 19.

Sample amount

Samples of 10^2–10^7 cultured eukaryotic cells or 0.5–250 mg of tissue can be processed per prep. However, if the initial lysate volume exceeds 300 µL, the samples will need to be processed in multiple loads due to limitations of filter capacity.

D. Cell Lysis and Tissue Disruption

1. Collect 10^2–10^7 cells or 0.5–250 mg tissue; wash cells in cold PBS

Cultured mammalian cells

Ideally cells in culture should be processed fresh (i.e. not frozen). If you need to store cells before RNA isolation, they can be stored in RNA*later*, or they can be pelleted, frozen in liquid nitrogen, and stored at –70°C or colder.

Suspension cells: Count the cells, then pellet 10^2–10^7 cells at low speed, and discard the culture medium. Wash the cells by resuspending in ~1 mL PBS, and repelleting. Place the washed cells on ice.

Adherent cells: do one of the following

- Aspirate and discard the culture medium, and rinse with PBS. Place the culture plate on ice.
- Trypsinize cells to detach them and count. Then inactivate the trypsin, pellet the cells, and discard the supernatant (following the method employed in your lab for the cell type). Wash the cells by gently resuspending in ~1 mL PBS, and pelleting at low speed. Place the cells on ice.

Tissue samples

For good yield of intact RNA, it is very important to obtain tissue quickly and to limit the time between obtaining tissue samples and inactivating RNases in step c below.

a. Harvest tissue and remove as much extraneous material as possible, for example remove adipose tissue from heart, and remove gall bladder from liver. Perfuse the tissue with cold PBS, if desired, to eliminate some of the red blood cells.

b. If necessary, quickly cut the tissue into pieces small enough for either storage or disruption. Weigh the tissue sample (for samples to be stored in RNA*later,* this can be done later).

c. Inactivate RNases by one of the following methods:
   - Drop the sample into RNA*later*—tissue must be cut to ≤0.5 cm in at least one dimension for good penetration of the RNA*later*.
   - Disrupt the sample in Lysis/Binding Buffer as described in step 2. *Fresh (unfrozen) tissue* on page 10.
   - Freeze the sample in liquid nitrogen—tissue pieces must be small enough to freeze in a few seconds. When the liquid nitrogen stops churning, it indicates that the tissue is completely frozen. Once frozen, remove the tissue from the liquid nitrogen and store it in an airtight container at –70°C or colder.

2. Disrupt samples in 300–600 µL Lysis/Binding Buffer

Cultured cells

a. Remove the PBS wash or the RNA*later*, and add 300–600 µL Lysis/Binding Solution for 100–10^7 cells. Cells will lyse immediately upon exposure to the Lysis/Binding Solution. Use the low end of the range (~300 µL) for small numbers of cells (hundreds), and use closer to 600 µL when isolating RNA from larger numbers of cells (thousands–millions).
   For adherent cells lysed directly in the culture plate, collect the lysate with a rubber spatula, and pipet it into a tube.

b. Vortex or pipet vigorously to completely lyse the cells and to obtain a homogenous lysate. Cell cultures typically do not require mechanical homogenization; however, it will not damage the RNA.
   Large frozen cell pellets (i.e. more than about 10^7 cells) may need to be ground to a powder as described for frozen tissue samples to isolate high quality RNA. Alternatively, samples may be transitioned to –20°C in RNA*later*-ICE (P/N AM7030) and processed as described in step 2.a above.

c. Proceed to section *II.E. Organic Extraction* on page 11.

Yeast or bacterial cultures

Use disruption techniques appropriate for yeast and bacterial cultures, which may require vigorous mechanical disruption. www.invitrogen.com/site/us/en/home/support/technical-support.html.

Solid tissue stored in Ambion RNA*later* Solution, or transitioned to –20°C in RNA*later*-ICE Solution

Samples in RNA*later* and RNA*later*-ICE solution can usually be homogenized by following the instructions for fresh tissue (below). Extremely tough/fibrous tissues in RNA*later* may need to be frozen and pulverized according to the instructions for frozen tissue in order to achieve good cell disruption.

Blot excess liquid from samples, and weigh them before following the instructions for fresh tissue below.

Fresh (unfrozen) tissue

IMPORTANT

Use at least a 1:10 ratio (w/v) of tissue to Lysis/Binding Buffer for all tissues.

a. Measure or estimate the weight of the sample.

b. Aliquot 10 volumes per tissue mass of Lysis/Binding Buffer into a homogenization vessel on ice (e.g. aliquot 1 mL Lysis/Binding Buffer for 0.1 g tissue).
   Keeping the sample cold, thoroughly disrupt the tissue in Lysis/Binding Buffer using a motorized rotor-stator homogenizer. A ground-glass homogenizer or a plastic pestle can be used for small pieces (≤10 mg) of soft tissue. Homogenize until all visible clumps are dispersed.

c. Proceed to section *II.E. Organic Extraction* on page 11.

Frozen tissue, and extremely hard tissues

(Frozen tissue transitioned to –20°C in RNA*later*-ICE: process as for fresh tissue). Once the tissue has been removed from the –70°C freezer, it is important to process it immediately without even partial thawing. This is necessary because as cells thaw, ice crystals rupture both interior and exterior cellular compartments, releasing RNases.

a. Measure or estimate the weight of the sample.

b. Place 10 volumes of Lysis/Binding Buffer per tissue mass into a plastic weigh boat or tube on ice.
   We suggest using a weigh boat because it is easier to transfer frozen powdered tissue to a weigh boat than to a tube of Lysis/Binding Buffer.

c. Grind frozen tissue to a powder with liquid nitrogen in a prechilled mortar and pestle sitting in a bed of dry ice.

d. Using a prechilled metal spatula, scrape the powdered tissue into the Lysis/Binding Buffer, and mix rapidly.

e. Transfer the mixture to a vessel for homogenization and process the mixture to homogeneity, i.e. until all visible clumps are dispersed. If available, use a motorized rotor-stator homogenizer (e.g. Polytron).

f. Proceed to section E. Organic Extraction below.

E. Organic Extraction

Include this organic extraction both for total RNA purification and for purification of small RNAs.

1. Add 1/10 volume of miRNA Homogenate Additive, incubate 10 min on ice

a. Add 1/10 volume of miRNA Homogenate Additive to the cell or tissue lysate (or homogenate), and mix well by vortexing or inverting the tube several times. For example, if the lysate volume is 300 µL, add 30 µL miRNA Homogenate Additive.

b. Leave the mixture on ice for 10 min.

2. Extract with a volume of Acid-Phenol: Chloroform equal to the initial lysate volume

a. Add a volume of Acid-Phenol:Chloroform that is equal to the lysate volume before addition of the miRNA Homogenate Additive. For example, if the original lysate volume was 300 µL, add 300 µL Acid-Phenol:Chloroform.

IMPORTANT

Be sure to withdraw from the bottom phase in the bottle of Acid-Phenol:Chloroform, because the upper phase consists of an aqueous buffer.

b. Vortex for 30–60 sec to mix.

c. Centrifuge for 5 min at maximum speed (10,000 x g) at room temperature to separate the aqueous and organic phases. After centrifugation, the interphase should be compact; if it is not, repeat the centrifugation.

3. Recover the aqueous phase; transfer the aqueous phase to a fresh tube

Carefully remove the aqueous (upper) phase without disturbing the lower phase, and transfer it to a fresh tube. Note the volume removed.

F. Final RNA Isolation

Choice of elution solution

At the end of this procedure, RNA can be eluted in either nuclease-free water or in the Elution Solution provided with the kit. Elution Solution is nuclease-free 0.1 mM EDTA, if this could interfere with your application, elute in nuclease-free water instead.

Pre-heat eluent to 95°C

Preheat Elution Solution or nuclease-free water to 95°C for use in eluting the RNA from the filter at the end of the procedure.

100% ethanol must be at room temperature

If the 100% ethanol you plan to use for this procedure is stored cold, warm it to room temperature before starting the Final RNA Isolation.

Separate procedures for large and small RNA isolation

Separate procedures are provided for isolating total RNA (section F.I below), or for isolating RNA that is highly enriched for RNAs smaller than ~200 bases (section F.II on page 13). Choose the procedure that is better suited to your needs.

IMPORTANT

To isolate RNA for miRNA expression profiling using miRNA arrays, we recommend following the procedure for total RNA isolation (not the enrichment procedure for small RNAs). This makes it possible to critically evaluate the quality of the RNA to verify that it is suitable for array analysis and to quantitate the RNA. The miRNA population should then be further purified (e.g., with the flashPAGE™ system) before labeling samples for miRNA array analysis.

F.I. Total RNA Isolation Procedure

1. Add 1.25 volumes 100% ethanol, and mix thoroughly

Add 1.25 volumes of room temperature 100% ethanol to the aqueous phase (e.g. if 300 µL was recovered in step E.3, add 375 µL ethanol).

2. Pass the lysate/ethanol mixture through a Filter Cartridge

a. For each sample, place a Filter Cartridge into one of the Collection Tubes supplied.

b. Pipet the lysate/ethanol mixture (from the previous step) onto the Filter Cartridge. Up to 700 µL can be applied to a Filter Cartridge at a time, for samples larger than this, apply the mixture in successive applications to the same filter.

c. Centrifuge for ~15 sec to pass the mixture through the filter. Centrifuge at RCF 10,000 x g (typically 10,000 rpm). Spinning harder than this may damage the filters.
Alternatively, vacuum pressure may be used to pass samples through the filter.

d. Discard the flow-through, and repeat until all of the lysate/ethanol mixture is through the filter. Reuse the Collection Tube for the washing steps.

3. Wash the filter with 700 µL miRNA Wash Solution 1

Apply 700 µL miRNA Wash Solution 1 (working solution mixed with ethanol) to the Filter Cartridge and centrifuge for ~5–10 sec or use a vacuum to pull the solution through the filter. Discard the flow-through from the Collection Tube, and replace the Filter Cartridge into the same Collection Tube.

4. Wash the filter twice with 500 µL Wash Solution 2/3

a. Apply 500 µL Wash Solution 2/3 (working solution mixed with ethanol) and draw it through the Filter Cartridge as in the previous step.

b. Repeat with a second 500 µL aliquot of Wash Solution 2/3.

c. After discarding the flow-through from the last wash, replace the Filter Cartridge in the same Collection Tube and spin the assembly for 1 min to remove residual fluid from the filter.

5. Elute RNA with 100 µL 95°C Elution Solution or Nuclease-free Water

Transfer the Filter Cartridge into a fresh Collection Tube (provided with the kit). Apply 100 µL of pre-heated (95°C) Elution Solution or nuclease-free water to the center of the filter, and close the cap. Spin for ~20–30 sec at maximum speed to recover the RNA.

Collect the eluate (which contains the RNA) and store it at –20°C or below.

F.II. Enrichment Procedure for Small RNAs

This variation of a traditional glass-fiber filter RNA purification yields RNA that is significantly enriched for small RNAs. This enrichment is accomplished by first immobilizing large RNAs on the filter with a relatively low ethanol concentration and collecting the flow-through containing mostly small RNA species. More ethanol is then added to this flow-through, and the mixture is passed through a second glass filter where the small RNAs are immobilized. This second filter is then washed a few times, and the small-RNA enriched sample is eluted.

1. Add 1/3 volume 100% ethanol, and mix thoroughly

Add 1/3 volume of 100% ethanol to the aqueous phase recovered from the organic extraction (e.g. add 100 µL 100% ethanol to 300 µL aqueous phase). Mix thoroughly by vortexing or inverting the tube several times.

2. Pass the sample through a Filter Cartridge, and collect the filtrate

a. For each sample, place a Filter Cartridge into one of the Collection Tubes supplied.

b. Pipet the lysate/ethanol mixture (from the previous step) onto the Filter Cartridge. Up to 700 µL can be applied to a Filter Cartridge at a time. For sample volumes greater than 700 µL, apply the mixture in successive applications to the same filter.

IMPORTANT

Collect the filtrate (that is, the liquid flow-through in the Collection Tube); it contains the small RNAs.

c. Centrifuge for ~15 sec to pass the mixture through the filter. Centrifuge at RCF 10,000 x g (typically 10,000 rpm). Spinning harder than this may damage the filters.
Alternatively, vacuum pressure can be used to pull samples through the filter.

d. Collect the filtrate. If the lysate/ethanol mixture is >700 µL, transfer ***the flow-through*** to a fresh tube, and repeat until all of the lysate/ethanol mixture is through the filter. Pool the collected filtrates if multiple passes were done, and measure the total volume of the filtrate.

NOTE

At this point, the filter contains an RNA fraction that is depleted of small RNAs. This fraction can be recovered if desired by treating the filter as described in steps F.I.3–5.

3. Add 2/3 volume 100% ethanol and mix thoroughly

Add 2/3 volume room temperature 100% ethanol to filtrate (i.e. flow-through). For example, if 400 µL of filtrate is recovered, add 266 µL 100% ethanol. Mix thoroughly.

4. Pass the mixture through a second Filter Cartridge, and discard the flow-through

a. For each sample, place a Filter Cartridge into one of the Collection Tubes supplied.

b. Pipet the filtrate/ethanol mixture (from the previous step) onto a second Filter Cartridge. Up to 700 µL can be applied to a Filter Cartridge at a time. For sample volumes greater than 700 µL, apply the mixture in successive applications to the same filter.

c. Centrifuge for ~15 sec to pass the mixture through the filter. Centrifuge at RCF 10,000 x g (typically 10,000 rpm). Spinning harder than this may damage the filters.
Alternatively, vacuum may be used to pass samples through the filter.

d. Discard the flow-through, and repeat until all of the filtrate/ethanol mixture is through the filter. Reuse the Collection Tube for the washing steps.

5. Wash the filter with 700 µL miRNA Wash Solution 1

Apply 700 µL miRNA Wash Solution 1 (working solution mixed with ethanol) to the Filter Cartridge and centrifuge for ~5–10 sec or use vacuum to pass the solution through the filter. Discard the flow-through from the Collection Tube, and replace the Filter Cartridge into the same Collection Tube.

6. Wash the filter twice with 500 µL Wash Solution 2/3

a. Apply 500 µL Wash Solution 2/3 (working solution mixed with ethanol) and draw it through the Filter Cartridge as in the previous step.

b. Repeat with a second 500 μL aliquot of Wash Solution 2/3.

c. After discarding the flow-through from the last wash, replace the Filter Cartridge in the same Collection Tube and spin the assembly for 1 min to remove residual fluid from the filter.

7. Elute RNA with 100 μL 95°C Elution Solution or Nuclease-free Water

Transfer the Filter Cartridge into a fresh Collection Tube (provided with the kit). Apply 100 μL of pre-heated (95°C) Elution Solution or nuclease-free water to the center of the filter, and close the cap. Spin for ~20–30 sec at maximum speed to recover the RNA.

Collect the eluate (which contains the RNA) and store it at –20°C or colder.

G. Analyzing RNA after Purification

RNA quantitation and quality assessment

Measuring the absorbance at 260 nm (A_{260}) in a spectrophotometer is a simple method for determining the concentration of RNA. The ratio of A_{260} to A_{280} provides an indication of RNA purity. See section IV.B on page 21 for detailed instructions on using spectrophotometer readings to assess RNA.

An alternative way to estimate RNA quality and quantity is to run a sample on a denaturing gel. To visualize small RNAs, use a denaturing acrylamide gel (see section IV.C on page 22). To visualize ribosomal RNA, run the samples in a denaturing agarose gel. www.invitrogen.com/site/us/en/home/support/technical-support.html.

Analyzing RNA for specific sequences

Traditional Northern blots are useful for the detection of relatively large RNAs, over ~100 bases. For small RNAs, however, samples must be run on denaturing acrylamide gels for good resolution, and these gels require a different transfer technique from ordinary agarose gel systems. We provide a brief procedure for Northern blotting to detect small RNAs in section IV.D on page 23.

Solution hybridization assays can provide an unrivaled degree of precision for detection and quantitation of small RNA molecules. In addition, with carefully designed probes, multiple targets can be assessed in the same assay. We offer an optimized system for miRNA detection in solution hybridization assays—the *mir*Vana miRNA Detection Kit (P/N AM1552). For available products, see our product catalog at www.invitrogen.com/ambion.

III. Troubleshooting

A. RNA Appears Degraded on Gel

1. Problems during electrophoresis

a. Ribosomal RNA (rRNA) is overloaded

When analyzing total RNA preps, running more than about 5 µg of RNA in a single lane may cause smiling and/or smearing of the rRNA bands. rRNA comprises about 80% of total RNA, so if 5 µg of total RNA is loaded in a gel lane, there will be about 1 µg and 3 µg of RNA in the 18S and 28S rRNA bands respectively. However, problems with too much rRNA should not be an issue for small RNA enriched samples.

b. Samples are incompletely denatured

Routine agarose gel analysis of the larger (mRNA, rRNA) species is typically done under denaturing conditions with either formaldehyde or glyoxal (both formaldehyde and glyoxal gel loading buffers are offered: P/N AM8552, AM8551, respectively). This requires heating the samples with the formaldehyde or glyoxal loading buffers for 15 min at 65°C, or 30 min at 50°C respectively.

To separate and visualize small RNAs, samples are run in acrylamide gels containing urea. For these gels (which are not normally used to examine total RNA) samples are denatured before loading in Gel Loading Buffer II, which contains formamide. To ***completely*** denature RNA, the sample should be diluted with an equal volume of Gel Loading Buffer II, then incubated in a 95°C water bath or heat block for at least 2 min.

2. RNA is degraded

a. Improper handling of tissue

It is extremely important to inactive RNases as quickly as possible after sample collection to avoid RNA degradation. It is also important to limit the time between death and sample processing for the best yield of high quality RNA.

b. Frozen tissue thawed before immersion in Lysis/Binding Solution

It is essential that frozen tissue stays frozen until it is disrupted in Lysis/Binding Solution.

If the tissue is frozen in small pieces (i.e. <0.5 cm^3), and it is to be processed with a motorized rotor-stator homogenizer, it can often be dropped directly into the Lysis/Binding Solution and processed before it has a chance to thaw. This shortcut generally only works for relatively soft tissues.

When powdering tissue in a mortar and pestle, it is important to prechill the mortar and pestle, and to keep adding small amounts of liquid nitrogen during grinding so that the tissue never thaws, even partially. Once the tissue is completely powdered, it should be mixed

with the Lysis/Binding Solution quickly before any thawing can occur. It may be convenient to scrape the frozen powder into a plastic weigh boat containing 10 volumes of Lysis/Binding Solution.

As an alternative to grinding frozen samples with liquid nitrogen, frozen samples can be treated with RNA*later*-ICE (P/N AM7030, AM7031) to render them malleable while keeping the RNA intact.

c. **Exogenous RNase contamination**
Once the lysate is bound to the filter, and the Lysis/Binding Solution is removed by the washing steps; all the typical precautions against RNase contamination should be observed. Gloves should be worn at all times, and changed frequently to avoid the introduction of "finger RNases". The bags containing the Collection Tubes, and the solution bottles should be kept closed when they are not in use to avoid contamination with dust. Any tubes or solutions not supplied with the kit, which will come in contact with the RNA, should be bought or prepared so that they are free from RNases.

For more information, go to www.invitrogen.com/site/us/en/home/support/technical-support.html.

B. RNA Yield Is Lower than Expected or Inconsistent

1. Poor tissue disruption

Rapid, thorough tissue disruption is one of the most important criteria for obtaining a high yield of RNA. Often, the best way to disrupt tissue is by grinding frozen tissues in liquid nitrogen, and then homogenizing using a rotor-stator type homogenizer. It is useful with very tough tissues to break the frozen tissue with a hammer before attempting to crush it in a mortar and pestle. In some cases, it may be impossible to completely disrupt the tissue. For example, due to its high content of connective tissue, breast tumor tissue is not amenable to either complete homogenization of fresh minced tissue or to thorough crushing of frozen tissue in liquid nitrogen. In such cases it may be advisable to go ahead with the isolation procedure after a reasonable effort at disruption, even if the tissue appears to still be mostly or partially intact.

Tissues can be rendered amenable to homogenization without either thawing or grinding by using RNA*later*-ICE.

For more information, go to www.invitrogen.com/site/us/en/home/support/technical-support.html.

2. Tissue or cells contain less RNA than expected

Expected yields of RNA vary widely between tissues. Researchers accustomed to working with tissues such as liver or kidney where RNA is plentiful may have unrealistically high expectations of RNA yields from tissues such as muscle, lung, or brain. Specific miRNAs also have a characteristic tissue distribution, and some tissues are devoid of detectable levels of miRNAs.

C. Contaminants in RNA; RNA Inhibits Enzymatic Reactions

1. DNA contamination

Digest the prep with DNase I (RNase-free)

Contaminating DNA can be removed by DNase digestion, with subsequent removal of DNase and buffer from the sample. We recommend DNA-*free*™ (P/N AM1906), which is formulated specifically for this type of application.

2. Other contaminants

Any of the following strategies will further purify RNA.

a. Digest with 100 µg/mL Proteinase K in the presence of 0.5% SDS at 37°C for 30 min. Extract with an equal volume of phenol/chloroform. Concentrate the RNA by ethanol precipitation as described below.

b. Ethanol precipitate the sample RNA as follows: Add ammonium acetate to a final concentration of 2–2.5 M. Mix well and then add 4 volumes of ethanol, and mix again. Chill at –20°C or lower for at least 30 min, then centrifuge at 16,000 x g for 20–30 min to pellet the RNA. Wash the pellet twice with 80% ethanol. Resuspend the RNA in nuclease-free water or TE (10 mM Tris-HCl pH 8, 1 mM EDTA).

c. Repeat the glass-fiber filter purification. Add 1/10th volume of 5M NaCl and 1.38 volumes of ethanol to the sample before repassing the sampleover a fresh Filter Cartridge. Continue the total RNA purification from step F.2 on page 12.

IV. Additional Procedures

A. Isolation of Small RNAs from Total RNA Samples

The enrichment procedure for small RNAs described in section II.F.II starting on page 13 can be used to enrich total RNA samples for the small RNA fraction (<200 nt). The total RNA sample is simply mixed with Lysis/Binding Buffer and miRNA Homogenate Additive, and is sequentially bound to two filters without phenol extraction. The end product is two fractions enriched either in large (first filter) or small (second filter) RNA species that can be used in independent experiments.

Preheat Elution Solution to 95°C:
Preheat the Elution Solution to 95°C for use in eluting the small RNA from the filter at the end of the procedure (step IV.A.9).

1. Mix total RNA with 5 volumes Lysis/Binding Buffer

Mix 50–100 µg of total RNA with 5 volumes of Lysis/Binding Buffer.

(For example if the RNA sample volume is 40 µL, add 200 µL Lysis/Binding Buffer.)

If the RNA sample volume is <30 µL, add water to bring the sample to 30 µL, then add 150 µL of Lysis/Binding Solution.

2. Add 1/10 volume of miRNA Homogenate Additive, incubate 10 min on ice

a. Add 1/10 volume of miRNA Homogenate Additive to the RNA mixture from the previous step, and mix well by vortexing or inverting the tube several times.
(For example, if the RNA sample mixture from the previous step is 240 µL, add 24 µL of miRNA Homogenate Additive.)

b. Leave the mixture on ice for 10 min.

3. Add 1/3 volume 100% ethanol, and mix thoroughly

Add 1/3 volume of 100% ethanol to the RNA mixture from the previous step (e.g. add 88 µL 100% ethanol to the 264 µL RNA mixture example from the previous step). Mix thoroughly by vortexing or inverting the tube several times.

4. Pass the mixture through a Filter Cartridge, and collect the filtrate

a. For each sample, place a Filter Cartridge into one of the Collection Tubes supplied.

b. Pipet the lysate/ethanol mixture (from the previous step) onto the Filter Cartridge. Up to 700 µL can be applied to a Filter Cartridge at a time. For sample volumes greater than 700 µL, apply the mixture in successive applications to the same filter.

IMPORTANT

Collect the filtrate (that is, the liquid flow-through in the Collection Tube); it contains the small RNAs.

c. Centrifuge for ~1 min to pass the mixture through the filter. Centrifuge at RCF 5,000 x g (typically 5,000 rpm).
Alternatively, vacuum pressure can be used to pull samples through the filter.

d. Collect the filtrate. If the RNA mixture is >700 μL, transfer ***the flow-through*** to a fresh tube, and repeat until all of the RNA mixture is through the filter. Pool the collected filtrates if multiple passes were done, and measure the total volume of the filtrate.

NOTE

At this point, the filter contains an RNA fraction that is depleted of small RNAs. This fraction can be recovered if desired by treating the filter as described in steps II.F.3–5 on page 13.

5. Add 2/3 volume 100% ethanol to the filtrate, and mix thoroughly

Add 2/3 volume room temperature 100% ethanol to filtrate (i.e. flow-through). For example, if 400 μL of filtrate is recovered, add 266 μL 100% ethanol. Mix thoroughly.

6. Pass the filtrate/ethanol mixture through a second Filter Cartridge, and discard the flow-through

a. For each sample, place a Filter Cartridge into one of the Collection Tubes supplied.

b. Pipet the filtrate/ethanol mixture (from the previous step) onto a second Filter Cartridge. Up to 700 μL can be applied to a Filter Cartridge at a time. For sample volumes greater than 700 μL, apply the mixture in successive applications to the same filter.

c. Centrifuge for ~1 min to pass the mixture through the filter. Centrifuge at RCF 5,000 x g (typically 5,000 rpm).
Alternatively, vacuum may be used to pass samples through the filter.

d. Discard the flow-through, and repeat until all of the filtrate/ethanol mixture is through the filter. Reuse the Collection Tube for the washing steps.

7. Wash the filter with 700 μL miRNA Wash Solution 1

Apply 700 μL miRNA Wash Solution 1 (working solution mixed with ethanol) to the Filter Cartridge and centrifuge for ~1 min at RCF 5,000 x g, or use vacuum to pass the solution through the filter. Discard the flow-through from the Collection Tube, and replace the Filter Cartridge into the same Collection Tube.

8. Wash the filter twice with 500 μL Wash Solution 2/3

a. Apply 500 μL Wash Solution 2/3 (working solution mixed with ethanol) and draw it through the Filter Cartridge as in the previous step.

b. Repeat with a second 500 μL aliquot of Wash Solution 2/3.

c. After discarding the flow-through from the last wash, replace the Filter Cartridge in the same Collection Tube and spin the assembly for 1 min at RCF 10,000 x g to remove residual fluid from the filter.

9. Elute RNA from the filter with 2 x 50 μL hot Elution Solution

a. Transfer the Filter Cartridge into a fresh Collection Tube (provided with the kit). Apply 50 μL of 95°C Elution Solution, and close the cap.

b. Incubate at room temperature for ~2 min.

c. Spin for 1 min at RCF 10,000 x g to recover the RNA. Collect the eluate (which contains the RNA enriched for the small RNA fraction) and store it at –20°C or colder.

d. Repeat steps 9.a–c with a second aliquot of preheated Elution Solution. Collect the small RNA into the same Collection Tube.

B. Assessing RNA Concentration and Purity by UV Absorbance

Dilute the sample 1:50 to 1:500 in water to bring the concentration into the linear range of the spectrophotometer. Blank the spectrophotometer with water. Read the absorbance at 260 nm and 280 nm. Quantitate as discussed below or assess the RNA purity by comparing A_{260}/A_{280}.

Approximate quantitation

After determining the A_{260} value by multiplying the spectrophotometer reading by the dilution factor, the RNA concentration can be calculated with either of the following equations:

$$C\ (\text{mol/L}) = A_{260}/(\varepsilon \times l)$$

$$C\ (\mu\text{g/mL}) = \frac{A_{260}}{\varepsilon \times l} \times M \times 1000 = \frac{1000 \times M}{\varepsilon \times l} \times A_{260}$$

Where:

- ε = extinction coefficient ($L \times mol^{-1} \times cm^{-1}$)
- l = path length (cm): all modern spectrophotometers have a path length of 1 cm.
- M = molecular weight (g/mol)
- C = concentration

For short nucleic acids (<200 nt), $1000 \times M/\varepsilon \times l \approx 33$, the approximate concentration can be determined with the following formula:

Small RNA <200 nt: C (μg/mL) $\approx 33 \times A_{260}$

Total RNA: C (μg/mL) $\approx 40 \times A_{260}$

Assess purity of RNA

The purity of the RNA can be assessed from the ratio of A_{260}/A_{280}. For highly pure RNA a ratio of 1.8–2.1 is expected.

C. Gel Analysis of Small RNAs

The best way to quickly analyze small RNAs is to run an aliquot of the prep on a denaturing 15% polyacrylamide gel.

Prepare gel and solutions

10X TBE

TBE is generally used at 1X final concentration for preparing gels and/or for gel running buffer.

Do not treat TBE with diethylpyrocarbonate (DEPC).

Concentration	Component	for 1 L
0.9 M	Tris base	109 g
0.9 M	Boric Acid	55 g
20 mM	0.5 M EDTA	40 mL

Dissolve with stirring in about 850 mL nuclease-free water. Adjust the final volume to 1 L.

Alternatively, we offer 10X TBE as a ready-to-resuspend mixture of ultrapure molecular biology grade reagents (P/N AM9863). Each packet makes 1 L of 10X TBE.

Denaturing acrylamide gel

The following instructions are to prepare 15 mL of gel mix for 15% polyacrylamide gel with 8 M urea. 15 mL is enough gel solution for one 13 x 15 cm x 0.75 mm gel. For available products, see our product catalog at www.invitrogen.com/ambion.

Amount	Component
7.2 g	Urea
1.5 mL	10X TBE
5.6 mL	40% acrylamide (acryl:bis acryl = 19:1)
Add nuclease-free water to 15 mL	
Stir to mix, then add:	
75 µL	10% ammonium persulfate
15 µL	TEMED
Mix briefly after adding the last two ingredients, and pour gel immediately.	

Run sample on gel

1. Mix 1–2 µg of the RNA with an equal volume Gel Loading Buffer II.
2. Heat sample for 2–5 min at 95–100°C.
3. Load the sample on a denaturing 15% polyacrylamide gel and electrophorese at 30–45 mA.
4. Stop electrophoresis when the bromophenol blue dye front has migrated to the bottom of the gel.

5. Soak the gel for 5 min in a 0.5–1 µg/mL solution of ethidium bromide in 1X TBE.
6. Wash the gel for 2–5 min in 1X TBE.
7. Visualize the RNA using a UV transilluminator. High quality small RNA will have clearly visible tRNA, 5S rRNA, and 5.8S rRNA bands (Figure 2 and 3 on page 3).

D. Detection of Small RNAs by Northern Blot

Northern blots can provide qualitative and quantitative data on miRNA samples. The procedure of Patterson and Guthrie for prehybridization, hybridization, and washing (Patterson 1987) is summarized here.

1. Run sample on acrylamide gel

Run 2 µg of total RNA, or 1 µg miRNA enriched sample on denaturing acrylamide gel (see section IV.C on page 22 for instructions on gel electrophoresis of small RNAs). Stain the gel in ethidium bromide, and examine it on a transilluminator to make sure that there is good separation of the RNA.

2. Transfer RNA to membrane

After staining, transfer the RNA to a nylon membrane by electroblotting (We offer BrightStar®-Plus Nylon membrane P/N AM10100). This procedure can be performed in a semi-dry apparatus using a stack of three sheets of blotting paper soaked in 0.25X TBE placed above and below the gel/membrane. For 0.75 mm gels, transfer at 200 mA for at least 0.2 A-hr (A x hr). Extending this time does not result in loss of sample. After blotting, keep the membrane damp. Finally, UV crosslink the RNA to the membranes using a commercial crosslinking device (120 mJ burst over 30 sec).

3. Prepare probe

Specific miRNAs can be detected on Northern blots using 5' end-labeled antisense DNA probes. Typically several different probes can be used in a single hybridization reaction for detection of several targets of different sizes. Small (≤40 nt) RNA probes can alternatively be used to detect small RNAs. We offer the mirVana Probe and Marker Kit for optimized end labeling and clean-up of short probes for miRNA. For more information, go to www.invitrogen.com/ambion.

4. Prepare solutions

50X Denhardt's Solution

Amount	Component
10 g	Ficoll 400
10 g	bovine serum albumin
10 g	polyvinylpyrrolidone
to 1 L	nuclease-free water

20X SSC

Amount	Component
175.3 g	NaCl
88.2 g	sodium citrate
800 mL	nuclease-free water
pH to 7.0 with HCl	
to 1 L	nuclease-free water

5. Prehybridize ≥1 hr at 65°C

Prehybridize membrane in 10 mL prehybridization solution for at least 1 hr at 65°C. After prehybridizing, remove and discard prehybridization solution.

Northern blot prehybridization solution

Amount	Component
6X	SSC
10X	Denhardt's solution
0.2%	SDS

6. Hybridize 8–24 hr at room temp

Add 10 mL of hybridization solution. Hybridize the blot in ~10 mL of Northern blot hybridization solution containing $\geq 4 \times 10^5$ cpm 5' end-labeled antisense probe(s) for 8–24 hr with gentle agitation at room temp.

Northern blot hybridization solution

Amount	Component
6X	SSC
5X	Denhardt's solution
$1–5 \times 10^6$ cpm	5' end-labeled antisense probes
0.2%	SDS

Filter solution with 0.45 µm pore filter before use to remove particulates.

7. Wash the membrane 3 times at room temp

After hybridization, remove hybridization solutions.

Northern wash solution

Amount	Component
6X	SSC
0.2%	SDS

a. Wash blot with ~10 mL wash solution. Agitate at room temp for 5 min. Remove and discard wash solution.

b. Repeat the above wash 2 more times.

8. Wash the membrane once at 42°C

Wash once at 42°C (or ~10°C lower than estimated T_m of probe); add ~10 mL wash solution. Agitate at 42°C for 5–10 min. Remove and discard wash solution.

9. Expose to film

After the final wash, wrap the blots in plastic wrap and expose to x-ray film or a phosphorimager screen according to the manufacturer's instructions. The latter method allows quantification of the amount of signal present in each band.

10. Detecting larger RNAs in sample

To detect larger RNAs (e.g., mRNAs which are not resolved on an acrylamide gel) in the same samples, a second Northern blot can be made from an agarose gel system (e.g. Ambion NorthernMax® Kits P/N AM1940, AM1946). To hybridize the blot, antisense RNA probes can be transcribed from your own templates or templates we supply (P/N AM7675, AM7431, AM7423).

V. Appendix

A. References

Boom R, Sol CJA, Salimans MMM, Jansen CL, Wertheim-Van Dillen PME, and Van Der Noordaa J (1990) Rapid and Simple Method for Purification of Nucleic Acids. *Journal of Clinical Microbiology.* **28.3**: 495–503.

Chomczynski P and Sacchi N (1987). Single-step Method of RNA Isolation by Acid Guanidinium Thiocyanate-Phenol-Chloroform Extraction. *Analytical Biochem.* **162**: 156–159.

Patterson B and Guthrie C (1987). An essential yeast snRNA with a U5-like domain is required for splicing in vivo. *Cell.* **49**(**5**):613–24.

Zeng Y, Yi R, Cullen BR (2003). MicroRNAs and small interfering RNAs can inhibit mRNA expression by similar mechanisms. *Proc Natl Acad Sci U S A.* **100**(**17**):9779–84.

B. Quality Control

Functional Testing

Mouse liver RNA is prepared according to the kit instructions. The RNA is checked for integrity and minimum yield requirements by Northern blot analysis using a probe for the leT-7 miRNA.

Nuclease testing

Relevant kit components are tested in the following nuclease assays:

RNase activity
Meets or exceeds specification when a sample is incubated with labeled RNA and analyzed by PAGE.

Nonspecific endonuclease activity
Meets or exceeds specification when a sample is incubated with supercoiled plasmid DNA and analyzed by agarose gel electrophoresis.

Exonuclease activity
Meets or exceeds specification when a sample is incubated with labeled double-stranded DNA, followed by PAGE analysis.

C. Safety Information

WARNING

GENERAL SAFETY. *Using this product in a manner not specified in the user documentation may result in personal injury or damage to the instrument or device. Ensure that anyone using this product has received instructions in general safety practices for laboratories and the safety information provided in this document.*

- *Before using an instrument or device, read and understand the safety information provided in the user documentation provided by the manufacturer of the instrument or device.*

- *Before handling chemicals, read and understand all applicable Safety Data Sheets (SDSs) and use appropriate personal protective equipment (gloves, gowns, eye protection, etc). To obtain SDSs, see the "Documentation and Support" section in this document.*

1. Chemical safety

WARNING

GENERAL CHEMICAL HANDLING. *To minimize hazards, ensure laboratory personnel read and practice the general safety guidelines for chemical usage, storage, and waste provided below, and consult the relevant SDS for specific precautions and instructions:*

- *Read and understand the Safety Data Sheets (SDSs) provided by the chemical manufacturer before you store, handle, or work with any chemicals or hazardous materials. To obtain SDSs, see the "Documentation and Support" section in this document.*
- *Minimize contact with chemicals. Wear appropriate personal protective equipment when handling chemicals (for example, safety glasses, gloves, or protective clothing).*
- *Minimize the inhalation of chemicals. Do not leave chemical containers open. Use only with adequate ventilation (for example, fume hood).*
- *Check regularly for chemical leaks or spills. If a leak or spill occurs, follow the manufacturer's cleanup procedures as recommended in the SDS.*
- *Handle chemical wastes in a fume hood.*
- *Ensure use of primary and secondary waste containers. (A primary waste container holds the immediate waste. A secondary container contains spills or leaks from the primary container. Both containers must be compatible with the waste material and meet federal, state, and local requirements for container storage.)*
- *After emptying a waste container, seal it with the cap provided.*
- *Characterize (by analysis if necessary) the waste generated by the particular applications, reagents, and substrates used in your laboratory.*
- *Ensure that the waste is stored, transferred, transported, and disposed of according to all local, state/provincial, and/or national regulations.*
- ***IMPORTANT!*** *Radioactive or biohazardous materials may require special handling, and disposal limitations may apply.*

2. Biological hazard safety

WARNING

Potential Biohazard. *Depending on the samples used on the instrument, the surface may be considered a biohazard. Use appropriate decontamination methods when working with biohazards.*

WARNING

BIOHAZARD. *Biological samples such as tissues, body fluids, infectious agents, and blood of humans and other animals have the potential to transmit infectious diseases. Follow all applicable local, state/provincial, and/or national regulations. Wear appropriate protective equipment, which*

includes but is not limited to: protective eyewear, face shield, clothing/lab coat, and gloves. All work should be conducted in properly equipped facilities using the appropriate safety equipment (for example, physical containment devices). Individuals should be trained according to applicable regulatory and company/institution requirements before working with potentially infectious materials. Read and follow the applicable guidelines and/or regulatory requirements in the following:

In the U.S.:

- *U.S. Department of Health and Human Services guidelines published in Biosafety in Microbiological and Biomedical Laboratories found at: www.cdc.gov/biosafety*
- *Occupational Safety and Health Standards, Bloodborne Pathogens (29 CFR§1910.1030), found at: www.access.gpo.gov/nara/cfr/waisidx_01/29cfr1910a_01.html*
- *Your company's/institution's Biosafety Program protocols for working with/handling potentially infectious materials.*
- *Additional information about biohazard guidelines is available at: www.cdc.gov*

In the EU:

Check local guidelines and legislation on biohazard and biosafety precaution and refer to the best practices published in the World Health Organization (WHO) Laboratory Biosafety Manual, third edition, found at: www.who.int/csr/resources/publications/biosafety/WHO_CDS_CSR_LYO_2004_11/en/

VI. Documentation and Support

A. Obtaining SDSs

Safety Data Sheets (SDSs) are available from:

www.invitrogen.com/sds

or

www.appliedbiosystems.com/sds

Note: For the SDSs of chemicals not distributed by Life Technologies, **contact the chemical manufacturer.**

B. Obtaining support

For the latest services and support information for all locations, go to:

www.invitrogen.com

or

www.appliedbiosystems.com

At the website, you can:

- Access worldwide telephone and fax numbers to contact Technical Support and Sales facilities
- Search through frequently asked questions (FAQs)
- Submit a question directly to Technical Support
- Search for user documents, SDSs, vector maps and sequences, application notes, formulations, handbooks, certificates of analysis, citations, and other product support documents
- Obtain information about customer training
- Download software updates and patches

1560MC

Headquarters
5791 Van Allen Way | Carlsbad, CA 92008 USA
Phone +1 760 603 7200 | Toll Free in USA 800 955 6288
For support visit www.appliedbiosystems.com/support

www.lifetechnologies.com

life technologies™
